# Mapping and Validation of scRNA-Seq-Derived Cell-Cell Communication Networks in the Tumor Microenvironment

**DOI:** 10.3389/fimmu.2022.885267

**Published:** 2022-04-28

**Authors:** Kate Bridges, Kathryn Miller-Jensen

**Affiliations:** ^1^ Department of Biomedical Engineering, Yale University, New Haven, CT, United States; ^2^ Department of Molecular, Cellular, and Developmental Biology, Yale University, New Haven, CT, United States; ^3^ Systems Biology Institute, Yale University, New Haven, CT, United States

**Keywords:** single-cell RNA-sequencing (scRNA-seq), cell-cell communication network, tumor microenvironment, immunotherapy, spatial profiling, single-cell secretomics, graph theory

## Abstract

Recent advances in single-cell technologies, particularly single-cell RNA-sequencing (scRNA-seq), have permitted high throughput transcriptional profiling of a wide variety of biological systems. As scRNA-seq supports inference of cell-cell communication, this technology has and continues to anchor groundbreaking studies into the efficacy and mechanism of novel immunotherapies for cancer treatment. In this review, we will highlight methods developed to infer inter- and intracellular signaling from scRNA-seq and discuss how they have contributed to studies of immunotherapeutic intervention in the tumor microenvironment (TME). However, a central challenge remains in validating the hypothesized cell-cell interactions. Therefore, this review will also cover strategies for integration of these scRNA-seq-derived interaction networks with existing experimental and computational approaches. Integration of these networks with imaging, protein secretion measurements, and network analysis and mathematical modeling tools addresses challenges that remain with scRNA-seq to enhance studies of immunosuppressive and immunotherapy-altered signaling in the TME.

## Introduction

Single-cell technologies provide the resolution necessary to study intercellular heterogeneity. Specifically, single-cell RNA-sequencing (scRNA-seq) permits whole-transcriptome profiling of individual cells (1), which supports identification of rare cell subpopulations (2), inference of complex gene-gene regulatory networks (3), and tracing of developmental lineages (4). Further, scRNA-seq facilitates inference of cell-cell communication by measuring expression of genes encoding for corresponding ligands, receptors, intermediate signaling proteins, and intracellular targets across interacting cell types and under homeostatic and diseased conditions.

While scRNA-seq has been applied to a broad range of biological contexts, it has become an important tool to probe the tumor microenvironment (TME). The TME is a heterogeneous and constantly evolving milieu made up of populations of different cell types, cell-surface and secreted signals, and extracellular matrix (5, 6). These different cell types include cancer cells, stromal cells including fibroblasts and endothelial cells, and infiltrating immune cells, although this list is not exhaustive (7). Immune cells further partition into innate and adaptive immune cells, which are responsible for carrying out non-specific and tumor antigen-specific mechanisms of host defense, respectively (8). Innate immune cell types in the tumor predominantly include macrophages, dendritic cells (DCs), and neutrophils, while adaptive immune cell types include T cells and B cells. From the perspective of developmental lineage, it is worth noting that innate and adaptive immune cells do not derive from respective progenitors. Hematopoietic stem cells, which give rise to all immune cells, diversify into myeloid and lymphoid cell lineages (9). While myeloid cells can differentiate into the macrophages, DCs, and granulocytes of the innate immune system, lymphoid lineage cells contribute to both arms of immunity. For example, T cells and natural killer (NK) cells derive from a common lymphoid progenitor, although NK cells are considered innate immune cells due to their lack of tumor antigen-specific cell surface receptors (10).

Tumor cells promote their own survival by establishing an immunosuppressive and tolerogenic microenvironment. Through interactions with neighboring immune cells, tumor cells can manipulate immune functions to avoid immunosurveillance and coordinate delivery of key nutrients for growth (11). For example, tumor cells can take advantage of macrophage plasticity to reprogram them toward an anti-inflammatory phenotype (12). Anti-inflammatory tumor-associated macrophages (TAMs) have been shown to promote production of growth factors and neovascularization (13), which contribute to tumor growth and metastasis. These TAMs can also actively suppress adaptive T cell responses (14). Additionally, tumor cells can directly communicate with T cells *via* “immune checkpoints” (15), which act as gatekeepers to prevent an antitumor immune response from being mounted. Tumor cells can further act as architects of the spatial organization of the TME, orchestrating restriction of activated tumor-infiltrating lymphocytes to the periphery of the tumor bulk (16). This behavior yields an immune-excluded TME, which drastically limits the tumor-killing capacity of infiltrating immune cells. Broadly, immune cell infiltration into the tumor falls into three categories: immune-excluded, immune-inflamed (i.e., widespread presence in the tumor), and immune-desert (i.e., few to no cells in the tumor) (17). These patterns in infiltration are generally more applicable to the distribution of lymphoid lineage cells in the tumor, particularly T cells, while myeloid cells are commonly more ubiquitously distributed. Overall, the TME represents a spatially organized community of dysregulated cells, with communication between them giving rise to emergent behaviors including immune evasion and tumor growth.

**Figure 1:**
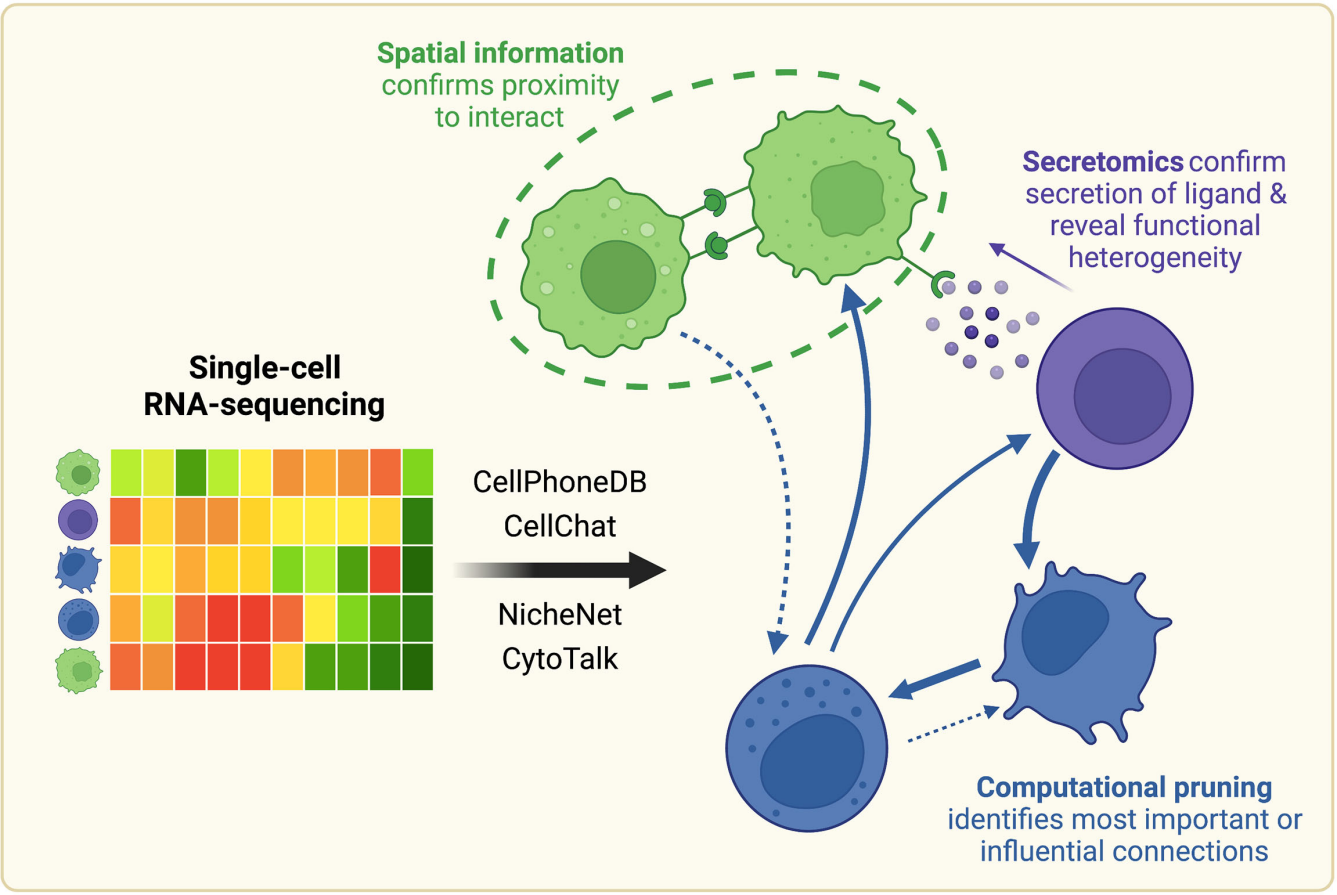
Once scRNA-seq-derived cell-cell communication networks are mapped, spatial profiling, protein-level measurements, and computational pruning can aid validation and interpretation. Cell-cell communication can be inferred from scRNA-seq using a variety of computational methods, such as CellPhoneDB, CellChat, NicheNet, or CytoTalk. Experimentally, connections between cell populations can be validated with spatial profiling and protein-level measurements. Networks can also be further analyzed computationally with network analysis and mathematical modeling tools. Created with BioRender.com.

Immunotherapy has emerged as a leading treatment option for advanced-stage tumors (18). By breaking immunosuppressive tumor-immune cell interactions and reestablishing anti-tumor immune signaling, this therapeutic option takes advantage of the body’s immune system to restore host defenses that were initially hijacked by the tumor. Immunotherapies have improved survival outcomes in numerous cancer types, and ongoing clinical trials serve to enhance the pool of effective interventions (19). Advancing our understanding of aberrant signaling in the TME directly supports the development of new immunotherapeutic strategies. Robust construction of hypothetical intercellular interaction networks from scRNA-seq is therefore critical to the design and testing of novel immunotherapeutic drugs that will ultimately continue to improve patient outcomes. This review will thus begin with an overview of existing computational methods for inference of communication between and within cells from scRNA-seq (**Figure 1**). Particular attention will be paid to their contributions to the fields of tumor immunology and immunotherapy development.

Despite these advances, a central challenge remains in the validation of the hypothesized cell-cell interactions. Computationally constructed communication networks often comprise hundreds to thousands of signaling interactions, which can make these networks impossible to probe experimentally in their entirety. Therefore, we will also review approaches to integrate these scRNA-seq-derived interaction networks with an array of existing experimental and computational methods to yield more meaningful biological conclusions (**Figure 1**).

## Initial Strategies to Infer Cell-Cell Communication From scRNA-Seq Focused on Matched Expression of Paired Receptor and Ligand

scRNA-seq affords the ability to measure expression of ligands and receptors to systematically decode how cells are communicating with one another. Evaluation of these interaction networks across homeostatic and diseased conditions facilitates identification of experimentally testable targets for tumor immunotherapy, among countless other applications. Many computational methods have been developed to examine cell-cell communication with scRNA-seq, and they exist along a spectrum with respect to their computational complexity and integration of additional signaling information. It should be noted that these algorithms require clustering of single cells into cell types or subsets of interest. There are many strategies for clustering and annotation of scRNA-seq data, which have been reviewed elsewhere (20–23).

Initial approaches generated hypotheses about cell-cell communication by quantifying matched expression of corresponding ligand and receptor. Zhou et al. (24) constructed the first cell-cell communication network of its kind from scRNA-seq collected from 19 patients with primary and metastatic melanoma (25). Interactions between malignant, immune, stromal, and endothelial cells were hypothesized by identifying paired receptor- and ligand-encoding genes from an author-curated list for which respective expression in two cell type populations was three standard deviations above the average across all populations. While the authors were then able to characterize tumor-immune, tumor-stromal, and tumor-endothelial cell crosstalk through specific signaling axes, they recognized the need to link this information with biological outcomes of interest, like tumor growth.

To address this limitation, Kumar et al. (26) developed a computational technique to quantitatively relate scRNA-seq-derived interaction networks from mouse and human metastatic melanomas to pathophysiological characteristics of the TME. They quantified cell-cell communication by calculating the product of average ligand and receptor expression across interacting cell type populations (referred to as interaction scores), with literature-supported ligand-receptor pairs sourced from the FANTOM5 database (27). To filter out interactions with inappropriately high scores due to noisy gene expression, the authors used statistical comparisons against null networks. Correlating interaction scores against tumor growth rate and anti-tumor immune response hypothesized ECM- and adhesion-related interactions, in addition to certain cytokine and chemokine interactions, as potential contributors to tumor progression in mice. This approach has thus helped to lay the foundation for identifying experimentally testable targets for immunotherapy from scRNA-seq-derived cell-cell communication networks.

## Emergence of Open-Source Tools for Interaction Network Construction From scRNA-Seq Has Advanced the Development of Cancer Immunotherapies

Despite the progress posed by the aforementioned methods, scalable application to existing scRNA-seq datasets remained a challenge as user-friendly platforms for construction of interaction networks did not exist. CellPhoneDB (28, 29) presented one of the first open-source tools for inference of cell-cell communication from single-cell transcriptomic data, which was originally applied to scRNA-seq from the human placenta. It quickly became one of the most utilized algorithms for the task, with their manuscripts amassing over 1,200 citations collectively since publication, and their online resource (www.cellphonedb.org) being used by over 500 users per month as of July 2020^1^. As with previously discussed approaches, CellPhoneDB probes cell-cell interactions by investigating matched expression of ligand-receptor pairs sourced from the UniProt (30, 31), Ensembl (32), Protein Data Bank (PDB) (33, 34), International Molecular Exchange (IMEx) consortium (35), and IUPHAR (36–38) databases. Critically, CellPhoneDB considers subunit architecture for both ligands and receptors, as opposed to the binary representation adopted by most methods.

CellPhoneDB has been widely used to understand cell-cell communication in the TME, particularly to characterize pro-tumor crosstalk between tumor, immune, and stromal cells. Macrophages are a type of immune cell that are especially vulnerable to exploitation by tumor cells because their activation is heavily context-dependent (39). Therefore, uncovering the signaling mechanisms by which tumor cells reprogram macrophages toward an anti-inflammatory pro-tumor phenotype, along with the mechanisms by which macrophages then carry out their pro-tumor behavior, will provide new targets for immunotherapeutic intervention. In both hepatocellular carcinoma (40) and esophageal squamous cell carcinoma (ESCC) (41), CellPhoneDB implicated the SPP1-CD44 signaling axis, among other receptor-ligand pairs, as a potential reprogramming interaction from tumor cells to macrophages. This axis has been previously implicated as an immune checkpoint in human cancers (42), and tumor cell signaling to macrophages and monocytes through the CD44 receptor has been shown to inhibit their anti-tumor response in *in vitro* co-culture models (43). In colorectal cancer (CRC), Zhang et al. (44) used CellPhoneDB to characterize the anti-inflammatory signaling from macrophages that had already shown evidence of pro-tumor reprogramming. Their analysis linked interactions involving SDC2, SPP1, and FN1 ligands between macrophages and cancer-associated fibroblasts, myofibroblasts, and endothelial cells to tumorigenesis and metastasis in CRC.

Beyond identifying potential therapeutic targets by characterizing pro-tumor signaling in the TME, CellPhoneDB has aided studies in understanding immunotherapy-altered signaling from single-cell transcriptomics. Immune checkpoint blockade (ICB) is the current standard of care for immunotherapeutic intervention for many cancers (45). Over time, anti-tumor effector immune cells in the tumor become “unresponsive” or “exhausted,” and become negatively regulated by ligands expressed in the TME. ICB aims to prevent or revert that immunosuppression by blocking these inhibitory interactions. One of the common targets of ICB is the programmed cell death protein 1 (PD-1) checkpoint. Signaling through PD-1 has been shown to negatively regulate T cell effector activity (46), and thus its blockade should limit the ability of tumor cells to evade immune surveillance and promote a successful anti-tumor response. However, mixed response rates to ICB in patients highlight the need for a deeper understanding of the mechanism of this therapy in the microenvironment. Studies in patients with breast cancer (47), advanced renal cell carcinoma (48), and basal cell carcinoma (49) have leveraged CellPhoneDB to characterize cell-cell communication associated with response and resistance to ICB. Across tumor types, this analysis identified signaling to CD8^+^ T cells *via* HAVCR2-LGALS9 (TIM3-Galectin9) as enhanced in non-responding and resistant patients. As TIM3 has been known to inhibit CD8^+^ cytotoxic activity and therefore can contribute to tumor progression (50), these studies emphasize the promise of CellPhoneDB to uncover conserved biomarkers for therapy resistance from scRNA-seq.

It is worth noting additional open-source packages for inference of cell-cell communication from scRNA-seq that use algorithms similar to CellPhoneDB. Network Analysis Toolkit for Multicellular Interactions (NATMI) (51) quantifies potential communication by calculating the product of average expression of matched ligand and receptor across two interacting cell type populations. The novelty of this method primarily derives from the curation of its reference database of ligand-receptor pairs, which borrows from competing methods and only includes pairs with primary literature support. NATMI does not consider subunit architecture for ligands or receptors. This method has been used to identify prognostic communication between tumor, stromal, and immune cells in patients with pancreatic ductal adenocarcinoma (PDAC) (52). CellTalker (53) probes matched expression of paired ligands and receptors from the FANTOM5 (27) database. This algorithm also does not take heteromeric complexes into account. While CellTalker does limit its analyses to genes that are differentially expressed across cell populations, which supports identification of population-specific signaling, it does not assign quantitative scores to potential interactions which restricts data-driven comparisons across conditions of interest. Finally, ICELLNET (54) also assigns interaction scores by multiplying the average expression of paired ligand and receptor, with an adjustment to this formula intended to account for receptor subunits. The ligand-receptor databases referenced by ICELLNET largely overlap with its competitors, including CellPhoneDB. This method has yet to be applied to investigate the TME in a published study.

## Functional Understanding of Cell-Cell Communication may Require More Than Paired Expression of Receptor and Ligand

CellPhoneDB in particular has advanced the study of intercellular signaling in the tumor microenvironment from scRNA-seq with its scalable and accessible platform. However, functional cell-cell communication extends beyond the interaction of extracellular ligand and receptor, as signaling cofactors can upregulate or antagonize downstream signal propagation, and ligands from “sender” cells can influence intracellular signaling cascades and target gene expression in “receiver” cells.

CellChat (55) is an algorithm for cell-cell communication inference from scRNA-seq that was developed to address the lack of modulation by signaling cofactors, such as soluble agonists, antagonists, and membrane-bound co-receptors, considered in previous methods. A mass action-based model underlies the interaction probability assigned to each ligand-receptor pair for each communicating set of cell populations. As opposed to inferring cell-cell communication directly from gene expression, CellChat first projects the transcriptomic profiles onto experimentally validated protein-protein interaction networks (56) using a network propagation approach (57). These projected profiles are then used to calculate interaction probability scores, with the scoring equation taking advantage of Hill functions to model positive and negative modulation of an interaction by signaling agonists and antagonists, respectively. This intermediate projection step is intended to account for the role of protein-protein interactions in ligand-receptor binding, which cannot be reflected in sparse gene expression profiles from scRNA-seq data without prior knowledge of how the proteins encoded by these genes can influence each other’s expression. Using the law of mass action addresses the fact that these protein-protein interactions can saturate.

First applied to the wound healing microenvironment, CellChat has shown promise in the characterization of malignant and immunotherapy-altered signaling in glioblastoma (58), esophageal squamous cell carcinoma (41), gastric cancer (59), and breast cancer (60). When compared directly to CellPhoneDB (55) across four mouse skin scRNA-seq datasets, CellChat achieved a slightly better true positive rate, a lower false positive rate, and higher accuracy. Accuracy in this context was defined as having more predictions that overlapped with the predictions of one or more methods, which in this study included the methods iTALK (61) (which has been published as a preprint) and SingleCellSignalR (62) in addition to CellPhoneDB. CellChat’s advantage over CellPhoneDB may also relate to the fact that this method limits its inquiries to genes that are differentially expressed (DEGs) across all cell groups in the data, as opposed to maintaining the transcriptome-wide gene set, which simplifies comparison across conditions. Including this same DEG-limiting step in the CellPhoneDB workflow, which was implemented in the newest version of the CellPhoneDB toolkit (CellPhoneDB v.3.0) (63), has improved its predictions as well. Further, CellChat and CellPhoneDB were found to similarly distinguish spatially adjacent from spatially distant interactions (55), unlike competing algorithms that only consider expression of paired receptor and ligand. This result supported the assertion that including subunit architecture and signaling cofactors serves as a proxy to help distinguish spatially relevant interactions from scRNA-seq. CellChat can also be distinguished from many of its competitors by its built-in tools for visualization and downstream analyses, which use concepts from graph theory, pattern recognition, and manifold learning to identify conserved and context-specific patterns across inferred signaling networks, among other important interpretations.

Other recently published algorithms NicheNet (64) and CytoTalk (65) have recognized the need to integrate intracellular signaling into scRNA-seq-derived communication networks. NicheNet was the first computational method to consider prior knowledge of intracellular signaling networks in its analysis of scRNA-seq by leveraging databases describing interactions between ligands, receptors, intracellular intermediaries, transcriptional regulators, and target genes. Strong correlation between expected patterns in gene expression, based on links between ligands and target genes in NicheNet’s prior model, and observed patterns in a user’s own scRNA-seq data identifies potential communication between interacting cell types. Similar to CellChat, NicheNet also takes advantage of DEGs to construct differential signaling networks across conditions of interest.

The use of a prior model with ligand-to-intracellular target gene axes to infer cell-cell communication from scRNA-seq data represented a departure from the interaction scoring approach matching ligand and receptor expression adopted by most preceding methods. Algorithmically, preceding methods follow a common procedure (**Table 1**, column 1). Taking annotated scRNA-seq data as input, they calculate interaction scores by combining average expression of ligand and receptor across two interacting cell populations with an elementary mathematical operation, generally multiplication. For example, Kumar et al.(26) and NATMI (51) rely on a simple product (**Table 1**, column 1, ii. A), while ICELLNET (54) includes receptor subunits in its formula (**Table 1**, column 1, ii. B). SingleCellSignalR (62) uses a regularized product, which accounts for total expression across cells (**Table 1**, column 1, ii. C). CellChat (55) technically also uses a regularized product, although its interaction scoring equation takes inferred protein expression as input as opposed to mRNA counts as competing algorithms do. Downstream analyses then generally focus on comparing these interaction scores statistically to null scores (to distinguish signal from noise) and across experimental conditions. As previously described, NicheNet instead correlates patterns in observed expression from annotated scRNA-seq data against expected expression in its prior model (**Table 1**, column 2). Expected expression of each gene in the prior model, given a particular stimulating ligand, is mathematically derived based on predicted propagation of signal from ligand to target gene through possible intermediate signaling genes. Downstream analyses with NicheNet generally further explore links between predicted ligands, receptors, and target genes in the prior model.

**Table 1:** Comparison of matched ligand-receptor (L-R) expression algorithms versus correlation against a prior model (e.g., NicheNet) for inference of cell-cell communication from scRNA-seq.

Matched L-R expression	Correlation against a prior model
i. Input: preprocessed scRNA-seq data annotated by cell type	i. Input: preprocessed scRNA-seq data annotated by cell type
ii. Calculate interaction score (IS) between cell types *j* and *k*, based on known ligand-receptor pairs (reference databases listed in Table 2), which takes on one of three general forms based on the method:	ii. Identify “target genes” in receiving cell populations (e.g., genes differentially expressed across conditions); remaining cells considered “background”
$$IS\left(L,R,j,k\right)=\{\begin{array}{c} L_{j}*R_{k},L_{j}+R_{k}\left(A\right)\\ \left(\sqrt[{{}^{z}}]{\prod_{z}L_{j,z}}\right)*\left(\sqrt[{{}^{m}}]{\prod_{m}R_{k,m}}\right)\\ \frac{\left(A\right)or\left(B\right)}{regularizationterm}\left(C\right) \end{array}\left(B\right)$$	iii. For each possible ligand *i*, calculate Pearson correlation (ρ_i_) between observed expression of target genes *j* and expected expression of *j* given *i* (E_i,j_) from prior model; ρ_i_ is penalized for correlations with background genes. E_i,j_ is defined as:
where $L_{j}=\left(\frac{1}{n_{j}}\sum_{i=1}^{n_{j}}l_{i}\right),R_{k}=\left(\frac{1}{n_{k}}\sum_{i=1}^{n_{k}}r_{i}\right),$	$E_{i,j}=\sum_{k=1}^{n}\left(PPR_{i,k})*(GRN_{k,j}\right)$
Or *L_j_*, *R_k_* are otherwise scaled representations of expression, e.g., z-scores, and *z* and *m* account for ligand and receptor subunits, respectively	where, based on the prior model, PPR_i,k_ denotes the probability of ligand *i* signaling through intermediate gene *k*, and GRN_k,j_ quantifies evidence that *k* regulates expression of target gene *j*. *n* is the number of possible intermediate genes *k*
iii. Downstream analyses: compare IS to null networks and across experimental conditions	iv. Downstream analyses: further explore ligand-target gene and ligand-receptor axes in the prior model

NicheNet has been cited over 200 times since its publication in 2020, with many of the citing papers investigating the TME. Cheng et al. (66) presented a prime example of NicheNet’s utility in studying myeloid signaling across tumor microenvironments. The authors investigated conventional dendritic cells (cDCs), mast cells, and tumor-associated macrophages (TAMs) using scRNA-seq data from 210 patients across 15 human cancer types. Following previous work from Maier et al. (67), cDCs were clustered across tumor types into two classical DC subsets (cDC1s and cDC2s) and a novel LAMP3^+^ mature cDC subset enriched for immunoregulatory genes (mregDCs). Cheng et al. were keen to understand mregDC development, as this novel DC subset has been shown to originate from both cDC1s and cDC2s, resulting in different roles in anti-tumor immunity. Using NicheNet, IL-4 and IL-12 were identified as drivers of the cDC1-derived mregDC signature, while IL-15 and IFNs were linked to the cDC2-derived mregDC signature (66). Modulating these NicheNet-identified signals in the TME has the potential to adjust the balance of cDC1- and cDC2-derived mregDCs in a way that supports an anti-tumor immune response. The authors also probed mast cells in the TME, as TNF^+^ and pro-angiogenic VEGFA^+^ mast cells have been thought to play dueling roles in the tumor (68). Both NicheNet and CellPhoneDB were used to uncover potential signaling underlying the TNF^+^ mast cell signature. Interestingly, while both methods implicated IL1β^+^ macrophages as the drivers of anti-tumor TNF^+^ mast cell behavior, the full suite of their top results offered little overlap. Whether the inclusion of intracellular signaling information in NicheNet’s method generates more accurate or biologically relevant hypotheses than algorithms like CellPhoneDB requires further experimental validation.

Although NicheNet advanced cell-cell communication inference from scRNA-seq by presenting a more mechanistic picture of interaction though ligand-to-target gene axes, its prior model does not include the additional prior knowledge that many signaling pathways are specific to a given cell type or tissue. Hu et al. (65) illustrated this heterogeneity in signaling downstream of fibroblast growth factor receptor 2 (FGFR2) between three pairs of interacting cell populations across mammary gland and skin. They therefore developed CytoTalk, which similarly constructs scRNA-seq-derived cell-cell communication networks through ligand-to-target gene axes, but with data-driven inference of signal transduction networks instead of a prior model. While NicheNet’s intracellular signaling networks were derived from published databases including OmniPath (69), CytoTalk constructs these networks *de novo* by quantifying relationships between genes from the user’s own data. CytoTalk has yet to be applied to the TME in a published study, although the utility of this method is clear considering the roles cell type and tissue specificity have been shown to play in signaling in cancer (70). Altogether, much progress has been made to extract increasingly accurate and biologically relevant information about cell-cell communication from scRNA-seq.

## Underlying Reference Databases Play a Key Role in the Inference of Cell-Cell Communication

It is worth mentioning that, across methods, predictions about cell-cell communication rely heavily on the underlying reference databases summarizing known ligand-receptor pairs and intracellular signaling pathways. Many of the aforementioned methods source interaction information from a similar pool of databases (**Table 2**, “Referenced databases”). The FANTOM5 database (27) specifically, which includes ~2,500 human ligand-receptor pairs, is referenced by nearly all of the selected algorithms in **Table 2**. Further comparison of the general features, advantages, and limitations of FANTOM5 against other commonly used reference databases, including those underlying CellPhoneDB, NATMI, ICELLNET, CellChat, iTALK, SingleCellSignalR, and NicheNet, has been reviewed elsewhere (89, 90).

**Table 2:** Selected summary of computational methods for inference of cell-cell communication.

Method	Application to TME?	Signaling components considered	Underlying mathematical model	Referenced databases	Primary advantages	Primary limitations*
Zhou et al. (24)	Melanoma	Paired ligand and receptor (L-R)	Matched significant upregulation of L and R	FANTOM5, DLRP (71), IUPHAR, HPMR (72), author additions	Laid foundation for interaction inference	Lacks other signaling components, statistical framework, links to emergent behaviors
Puram et al. (73)	Head and neck cancer	Paired L-R	Matched significant upregulation of L and R	FANTOM5	Laid foundation for interaction inference	Lacks other signaling components and statistical framework
Kumar et al. (26)	Melanoma	Paired L-R	Product of average L and R	FANTOM5, author additions	Analysis framework links interactions to emergent behaviors	Lacks other signaling components
Raredon et al. (74)	No	Paired L-R	Sum of average L and R	FANTOM5	Uses graph theory for cross-network comparison	Lacks other signaling components
CellPhoneDB (28, 29)	ESCC, CRC, breast cancer, among others	Paired L-R, including subunits	Matched significant upregulation of L and R	UniProt, Ensembl, PDB, IMEx, IUPHAR	Scalable, user-friendly platform; includes subunits	Lacks other signaling components
NATMI (51)	PDAC	Paired L-R	Product of average L and R	CellPhoneDB, SingleCellSignalR, ICELLNET, STRINGDB (56), RNA-magnet (75), author additions	Considers L-R specificity in interacting cell types; underlying database	Similar to predecessors, lacks other signaling components
CellTalker (53)	Head and neck cancer	Paired L-R	Matched expression of L and R	FANTOM5	Uses differentially expressed genes for focused exploration	Similar to predecessors, lacks other signaling components and statistical framework
ICELLNET (54)	No	Paired L-R, including receptor subunits	Product of average L and R	STRINGDB, Ingenuity, BioGRID (76), Reactome (77), CellPhoneDB	Includes receptor subunits	Similar to predecessors, lacks other signaling components
CellChat (55)	GBM, ESCC, breast cancer, among others	Paired L-R and signaling cofactors	Mass action-based model	KEGG (78), author additions	Includes signaling cofactors, uses graph theory for cross-network comparison	Lacks intracellular signaling information
iTALK (61)	Lung adeno-carcinoma (79)	Paired L-R	Matched significant upregulation of L and R	FANTOM5, DLRP, IUPHAR, HPMR, author curation of cytokine and chemokine interactions (80–82)	Author-curated reference interactions, built-in tools for visualization	Lacks other signaling components
SingleCellSignalR (62)	CRC (83), breast cancer (84), metastatic melanoma (85), among others	Paired L-R	Regularized product of average L and R	FANTOM5, HPMR, IUPHAR, UniProt, HPRD (86), GO (87), Reactome	Author-curated reference interactions	Lacks other signaling components
NicheNet (64)	Melanoma, CRC, breast cancer, among others	L-R and downstream intracellular signaling	Pearson correlation of user data with prior model	FANTOM5, IUPHAR, KEGG, OmniPath (69), MSigDB (88), among other PPI, gene regulatory interaction databases	Includes intracellular signaling components	Prior model doesn’t consider cell type or tissue specificity
CytoTalk (65)	No	L-R and intracellular signaling in both “sender” and “receiver”	Regularized sum of average L and R, mutual information between genes for inference of intracellular signaling	FANTOM5, author additions	Considers cell type and tissue specificity	Lacks extracellular signaling components (subunits, cofactors)

This column highlights limitations of communication inference methods when compared to each other. Limitations across methods (e.g., lack of spatial information) are discussed in the text.

Among many attributes of reference databases, there are two to consider when evaluating predictions from cell-cell communication inference methods. The first is the proportion of interactions in each database with primary literature evidence. For example, Hou et al. (51), in the manual curation of their own interaction database, excluded 94 ligand-receptor pairs from CellPhoneDB and 143 from the Human Protein Reference Database (HPRD) (86) for a lack of literature support. Ensuring primary experimental evidence for predicted interactions increases the accuracy and biological relevance of hypotheses generated by these cell-cell communication inference tools. The second consideration is the frequency with which these databases are updated with newly discovered or otherwise revised interactions. Whether a database of interactions reflects the current state of the literature further impacts a user’s interpretation of the predictions made by these methods on their own datasets. Altogether, there are many caveats to keep in mind when deriving cell-cell communication networks from scRNA-seq, with the underlying reference database of ligand-receptor and ligand-target gene axes being one of them.

## Shortcomings Across Existing Computational Methods Derive From Challenges That Remain With scRNA-Seq

scRNA-seq experiments require tissue dissociation (91, 92), which prevents the preservation of critical spatial information. Although the aforementioned computational methods (summarized in **Table 2**) leverage ligand-receptor pair databases and integrate critical information about intracellular and other relevant signaling, it remains difficult to overcome this key shortcoming in the method of data collection. Although some ligands are soluble, distances traveled by diffusion are limited; in other cases, both receptor and ligand are membrane-bound. Thus, inference of cell-cell communication would be greatly improved by incorporating knowledge of the physical proximity between two cell populations in the microenvironment. For example, tumors can disrupt successful immune responses by limiting T cell infiltration into their bulk (93), thus restricting the physical interaction between T cells and other cell types. In this case, integration of spatial information is crucial to filtering edges in scRNA-seq-derived communication networks describing these pathologies.

Additionally, heterogeneous signaling among broad cell types and cell subpopulations within a single cell type often differentially contribute to cancer progression. All cell-cell communication inference methods depend on how cell populations are defined. While clustering single cells into broad cell types has been extensively explored (20–23), subpopulation definition remains a contested topic. From scRNA-seq, unsupervised clustering of broader cell type lineages is often used to delineate cell subsets; this approach has been extensively applied to understand heterogeneity in the myeloid compartment of sarcoma (94) and non-small-cell lung cancer (95) tumors, among other malignancies (58, 66, 96, 97).

Because mRNA is not a functional measurement of the cell, defining cell subpopulations based on gene expression does not always separate cells into subsets that are relevant with respect to their functional signaling behavior. Directly measuring extracellular signaling events at the protein level partly overcomes this limitation. Single-cell secretomic technologies in particular have shown promise in the characterization of functional heterogeneity in the TME. For example, Perry et al. (98) used a single-cell secretion device to investigate myeloid heterogeneity in the melanoma TME, which identified specific functional subgroups of tumor-associated macrophages and myeloid cells (TAMMs) that aid orchestration of a pro-tumorigenic immune response. Inference of informative interaction networks from scRNA-seq thus also requires integration of protein-level measurements to confirm secretion of ligand and separate large cell-type populations into functionally relevant, context-dependent subpopulations.

Moreover, once the cell-cell communication networks are constructed, they need to be systematically compared across experimental conditions and interpreted with respect to emergent biological behaviors. As these networks are directed, weighted, and densely connected, data mining becomes an increasingly complex task. Therefore, this review will next discuss strategies for network validation and interpretation by leveraging imaging, protein secretion measurements, and network analysis and mathematical modeling tools. We anticipate that these integrations will aid generation of more biologically accurate and actionable hypotheses about aberrant and altered signaling mechanisms underlying tumor progression and as a result of immunotherapeutic intervention, respectively, from scRNA-seq.

## Spatial Information Is Critical to Confirming the Possibility of Interaction Between Cells in the TME

Because scRNA-seq requires tissue dissociation, the spatial information necessary to fully infer intercellular communication, particularly between membrane-bound receptors and ligands, is lost. Knowing the spatial localization of cells is especially critical to studies of the TME, as tumors can become “immune-excluded” (99), restricting the dialog between cancer and immune cells and rendering typical immunotherapeutic strategies useless. For example, T cell exclusion from the tumor bulk places crucial limits on the efficacy of T cell-targeted treatments, like ICB or adoptive cell transfer therapies. The field of spatially resolved transcriptomics (SRT) has begun to address the issue of a lack of spatial information by enabling confirmation of the physical proximity of cells expressing transcripts for a corresponding ligand and receptor pair. The advent of SRT has been particularly exciting for cancer research, yielding preliminary spatial maps of the prostate (100) and melanoma (101) TMEs, among others. With SRT, characterizing the TME *in situ* has suddenly evolved from low throughput “snapshots” given by immunohistochemistry and *in situ* hybridization to unbiased molecular profiling without compromising spatial context. For many of these reasons, SRT was named Nature Method’s “Method of the Year” for 2020 (102).

Due to the complexity of preserving spatial information, SRT technologies do lag behind scRNA-seq in depth of sequencing, resolution, and accessibility. Some SRT methods require a preselected panel of genes for profiling (103), while others have yet to achieve single-cell resolution. As opposed to replacing scRNA-seq, SRT methods thus present an opportunity to extract more biologically relevant hypotheses about cell-cell communication in the TME through integration with scRNA-seq. This is particularly important given the opportunity to further mine the plethora of scRNA-seq datasets available in the literature as a result of both independent studies and collaborative efforts like the Human Cell Atlas (104). Recently, several excellent in-depth reviews of SRT have been published (103, 105–107). Here our goal will be to highlight how these SRT methods have been integrated with scRNA-seq to enhance the investigation of interactions between cell populations in the TME. Moncada et al. (108) presented one of the first examples of the integration of the recently developed spatial transcriptomics (ST) method (109), capable of transcriptome-wide sampling but at low resolution, with scRNA-seq to understand the tissue architecture of pancreatic ductal adenocarcinoma (PDAC). With ST, the authors were able to identify patterns in spatially restricted enrichments and co-enrichments of tumor, myeloid, and healthy ductal cells with other cell types in the TME. Although the ST array cannot cover the entire tissue, nor does it achieve single-cell resolution, integration with scRNA-seq supported a focused exploration of the relationship between the co-localization of specific cell types and their potential to communicate in the PDAC TME.

Two other examples of SRT methods, multiplexed error-robust fluorescent *in situ* hybridization (MERFISH) (110, 111) and GeoMX Digital Spatial Profiling (DSP) (112), have aided studies of spatial organization in the TME. MERFISH is an imaging-based approach capable of generating single molecule resolution spatial maps of thousands of preselected genes (i.e., not transcriptome wide, a fundamental trade-off) at high detection efficacy. In glioblastoma (GBM), MERFISH revealed an enrichment of macrophages in the neighborhood of a specific subpopulation of malignant cells in both mouse models and patient samples (113). Integration of this information with scRNA-seq lent support for five ligand-receptor axes through which macrophages could be inducing a mesenchymal-like state in this GBM cell subtype. As mesenchymal-like GBM has been associated with more aggressive, invasive, and inflammatory features of the TME (114), the integration of MERFISH and scRNA-seq by Hara et al. directly resulted in the identification of targets for immunotherapeutic intervention in GBM. MERFISH has also been used to spatially map all cell types and states identified by scRNA-seq in CRC patient samples (115), linking spatial organization in the tumor to previously predicted cell-cell communication networks.

GeoMX DSP is an *in situ* barcoding method for multiplexed profiling of a predefined panels of RNAs or proteins from fixed tumor tissues (112). This method has been used to identify spatially informed biomarkers of response and resistance to immunotherapies such as ICB across multiple cancer types, including melanoma (116), non-small cell lung cancer (NSCLC) (117) and head and neck squamous cell carcinoma (HNSCC) (118). In melanoma and NSCLC, PD-L1 expression in the CD45^+^CD68^+^ spatial compartment (i.e., macrophages) was found to correlate with prolonged progression-free survival and overall survival in patients. Similar to MERFISH, GeoMX DSP thus provides high resolution imaging of focused regions of the TME, supporting local rather than global evaluations of ligand, receptor, and cell type co-localization.

It is worth noting that additional spatial profiling methods continue to be developed with potential to advance the study of the TME. Deterministic barcoding in tissue for spatial omics profiling (DBiT-seq) is a microfluidics-based method for 10μm-resolution multi-omic profiling of fixed tissues (119). By delivering barcodes to the surface of tissue slides, the authors were able to generate two-dimensional mosaics of protein and transcriptome-wide mRNA expression in mouse embryos. DBiT-seq specifically capitalized on the shortcomings associated with the technically demanding nature of *in situ* hybridization methods like MERFISH and sequential fluorescence *in situ* hybridization (seq-FISH+) (120) by taking advantage of next generation sequencing (NGS) technology and resources. Unlike GeoMX DSP and similar barcoding strategies [e.g., Slide-seq (121)], DBiT-seq uses microfluidics for guided delivery of barcodes to tissue, which greatly improves spatial accuracy. As of yet, DBiT-seq has not been used to map the spatial organization of a tumor in a published study, although it is likely only a matter of time due to the versatility and ease of use of this technology. The transcriptomic measurements captured by DBiT-seq would directly support inference of intercellular interactions at nearly (but not quite) single-cell resolution. DBiT-seq, like many SRT technologies, is still limited by pixel size and the total tissue area able to be mapped (119). Taken together, SRT methods, while not yet replacing scRNA-seq due to limitations in multiplexing and resolution, have and can continue to advance investigations of cell-cell communication in the TME when analyzed in conjunction with scRNA-seq.

Beyond SRT methods, an array of complementary imaging techniques for both dynamic and static receptor-ligand and sender-receiver cell co-localization has been developed and used in the TME. Intravital microscopy (IVM) (122) has emerged as a leading tool to visualize *in vivo* dynamics and migration in tumor models at single-cell resolution. Following surgical implantation of an imaging window, live fluorescent imaging of the TME can be collected, generally over a period of hours to days. Luthria et al. (123) showcased the utility of IVM in the identification of dynamic physical interactions between immune and tumor cells *in vivo* across multiple cancer models. TAMs were found to often neighbor and intertwine themselves around tumor cells, confirming physical interaction between the two cell populations. Moreover, the authors linked TAM polarization state to microtubule dynamics in tumor cells, as quantified by IVM, elucidating an association between the anti-inflammatory or pro-tumorigenic macrophage state and increased cancer cell migration and metastasis. Integration with scRNA-seq-derived communication networks generated hypotheses about the role that autocrine signaling (i.e., TAM-TAM interaction) played in anti-inflammatory TAM polarization. Using the cell-cell communication inference strategy from Kumar et al. (26) and scRNA-seq from patient biopsies across cancers, the authors implicated the interleukin 10 (IL10)-IL10R(A,B) axis as a critical feedback loop in TAMs that restricts their reprogramming toward a pro-inflammatory or anti-tumor phenotype (123). Treatment with an IL10R-blocking antibody was further found to stunt tumor cell motility and migration both *in vitro* (in a co-culture model system) and *in vivo*. The cell-cell interaction networks inferred from scRNA-seq therefore directly provided an experimentally testable target for immunotherapeutic intervention in this study, although the authors did primarily focus on crosstalk between immune cell populations in these hypothetical communication networks. Investigation of TAM-tumor cell crosstalk as inferred from scRNA-seq was not presented in this work. The focus of their IVM data was to demonstrate physical interactions between TAMs and tumor cells in the TME and pinpoint these interactions as a catalyst for downstream alterations in microtubule dynamics. Reanalysis of the scRNA-seq data to instead concentrate on inferring extracellular signaling events between TAMs and tumor cells has the potential to identify additional mechanisms of cancer cell migration and anti-inflammatory TAM polarization for therapeutic targeting with direct support from the IVM work.

Further, IVM has helped to elucidate the mechanism of anti-PD-1 checkpoint inhibition underlying effective anti-tumor responses (124). IVM revealed co-localization of CD8^+^ cytotoxic T cells and dendritic cells (DCs), producing IFNγ and IL-12 signals, respectively, upon treatment with anti-PD-1. This T cell-DC crosstalk was thus found to form a positive feedback loop resulting in stimulation of anti-tumor T cell immunity that was indirectly downstream of anti-PD-1 binding. Garris et al. (124) collected paired scRNA-seq from the MC38 colon carcinoma TME to support their claim that IL-12 production was exclusive to DCs, although inference of cell-cell communication from this data was not reported. Reanalyzing this scRNA-seq data to predict differential signaling between immune and tumor cells upon checkpoint inhibition could further the conclusions of this work by contextualizing the IFNγ/IL-12-mediated signaling in the greater network of cell-cell communication at play in the tumor, with support for communication between certain cell type populations coming from their IVM co-localization data. Other studies, which characterized crosstalk between immune cell populations (125) and between tumor cells and healthy tissue (126) in the TME with IVM, similarly collected paired scRNA-seq but did not present scRNA-seq-derived interaction network analysis. These works highlight existing opportunities in the field for reanalysis of publicly available transcriptomic data to infer and validate specific mechanisms and global signaling underlying emergent behaviors. IVM has also shown promise in studying the targeting (127, 128), dynamics, and effects on angiogenesis (129) of nanoparticle-based therapies for immunomodulation in the TME. This information would aid both validation and interpretation of therapy-altered communication networks inferred from scRNA-seq.

While IVM affords *in vivo* measurement of interacting cell co-localization and dynamics in the TME, this method is limited by the number of fluorophores that can be measured simultaneously. The need for an imaging window also makes IVM more suitable for collecting spatial information from animal models than humans, restricting the validation of cell-cell communication networks inferred from patient-derived scRNA-seq, although human intravital microscopy (HIVM) is under development (130). Albeit from fixed tumor slices, imaging methods such as tissue-based cyclic immunofluorescence (t-CyCIF) (131) can visualize a greater multiplicity of fluorophores simultaneously, permitting spatial mapping of cell type lineage markers, signaling proteins, drug targets, and immune cell antigens concurrently in the TME. Jerby-Arnon et al. (132) leveraged t-CyCIF in addition to scRNA-seq to define a relationship between T cell exclusion and therapy resistance in ICB-treated melanomas. Malignant cells expressing a resistance-associated gene program were often found to localize to T cell-depleted niches in the TME. As T cell exclusion has been recognized as a main player in the mixed patient responses to ICB, t-CyCIF can shed light on whether the spatial organization of a TME might support or hinder certain immunotherapeutic strategies. t-CyCIF has also aided studies of myeloid-targeted immunotherapies in breast cancer (133). Characterization of BRCA1-associated triple negative breast cancer (TNBC) tumors as highly infiltrated by immunosuppressive macrophages with t-CyCIF directly led the authors to explore therapies intended to reprogram these cells toward a more pro-inflammatory state in this TME. Integration with scRNA-seq-derived interaction networks would help to identify mechanisms by which macrophages orchestrate immunosuppression and provide auxiliary targets for immunotherapy.

Altogether, many strategies for spatial mapping of the TME exist, offering dynamic and static measurements of receptor-ligand and sender-receiver cell co-localization, *in vivo* and *ex vivo*. All have merit in the confirmation of physically possible interactions in scRNA-seq-derived communication networks, depending on the context-specific parameters of the TME being studied. Further, updating the aforementioned cell-cell communication inference methods to directly take spatial profiling information as input will enhance the integration of imaging with transcriptomics technologies. For example, as shown in their documentation^2^, CellPhoneDB v.3.0 (63) now allows the user to input additional information describing the localization of each cell type to different spatial niches in the microenvironment, as derived from prior knowledge, SRT, or imaging. Their algorithm then places restrictions on predicted interactions between cell types in different niches.

## Functional Signaling in the Tumor Microenvironment Can be Validated by Direct Measurement of Protein Secretion

mRNA expression does not provide a functional cellular measurement, nor does it generally correlate with protein levels (134), which fundamentally limits inference of protein signaling activity from scRNA-seq. Direct measurement of protein secretion is particularly critical when characterizing signaling in the tumor microenvironment, as many cytokine and chemokine networks modulated by immune cells are responsible for orchestrating pro- or anti-tumor effects. There are many commonly used platforms for protein measurement in the TME. Two of the most popular, fluorescence-activated cell sorting (FACS) and enzyme-linked immunosorbent assays (ELISA), maintain inherent limitations. Both are limited in their multiplexing capabilities, and FACS in particular measures surface markers and intracellular proteins as opposed to the secreted proteins of interest to signaling studies. Measuring protein secretion in the tumor microenvironment to complement scRNA-seq-derived communication networks requires immunophenotyping technology capable of higher throughput and multiplexing and that is tuned to capture secreted signals only.

The advent of microfabricated single-cell secretomic analysis devices has begun to clear some of the aforementioned hurdles (135–137). The assay platform presented in Lu et al. (138, 139) in particular has laid the foundation for investigating macrophage secretion *in vitro* and *ex vivo*. Their device, referred to as the single-cell barcode chip (SCBC), integrates subnanoliter microchambers with high-density antibody barcode microarrays for simultaneous detection of up to 15 cytokines from over a thousand single cells in parallel. Beyond fundamental studies of macrophage proinflammatory dynamics *in vitro* (140, 141), the SCBC has been leveraged to measure secretion directly *ex vivo* in tumor-associated myeloid cells and macrophages (i.e., TAMMs, which includes dendritic cells) isolated from 8-week-old murine melanoma tumors (98). Upon combinatorial treatment with two myeloid-targeted immunotherapies, CD40 agonist (CD40ag) and CSF1R blockade, a subset of TAMMs were found to upregulate their co-secretion of proinflammatory cytokines TNF, IL-6, IL-12, and chemokine CCL3, along with anti-inflammatory factor Chi3l3. Other TAMM subsets decreased their secretion of immunosuppressive proteins MMP9, CCL17, and CCL22 after treatment relative to control. Many of these cytokines and chemokines are known to modulate the behavior of other cell types in the TME through cell-cell communication. For example, IL-12 represents a key link between innate and adaptive immunity, as it has a potent effect on T cell activation and Th1-type responses through induction of IFNγ production (142). Further, defining cell subpopulations based on differences in functional signaling is a key component of disentangling biologically relevant interaction networks in the TME. Regarding scRNA-seq-derived cell-cell communication networks, integration with multiplexed measurement of cytokine and chemokine secretion, particularly of key inflammatory mediators like IL-12, from paired experimental conditions would help to validate hypothesized sender-population signaling at single-cell resolution.

While the SCBC has proven its utility in measuring secretion from tumor-associated myeloid cells (namely macrophages and DCs), an up-to-42-plex version of this device has been commercialized with IsoPlexis (www.isoplexis.com), and it has been primarily tuned to investigate T cell secretion. Commercialization has facilitated access to this technology by over 100 pharmaceutical companies and medical centers worldwide^3^. In particular, this T cell-tuned SCBC has been used to examine the relationship between T cell polyfunctionality and resistance to anti-PD-1 therapy in human patients across multiple cancer types (143, 144). Polyfunctionality in this context was defined as co-secretion of two or more cytokines. Co-secretion of effector proteins Granzyme B and IFNγ by CD4^+^ and CD8^+^ T cell subsets, as measured by the SCBC, was identified as a distinguishing factor between response and resistance to anti-PD-1. The commercialized device has also been used to understand the functional response to other T cell-targeted treatments, including chimeric antigen receptor (CAR) T cell therapy (145, 146). Altogether, microwell assay platforms like the SCBC have advanced functional measurement of immune cell signaling in the TME, which can greatly aid the validation of functional cell subsets and cell-cell communication inferred from scRNA-seq.

While these microwell devices provided marked advances regarding multiplexed measurement of protein secretion by single cells from the TME, they maintain two shortcomings. As with scRNA-seq, the aforementioned assays offer static, “snapshot” measurements of protein secretion, although multiple experiments could be conducted over a series of timepoints. Further, platforms like the SCBC require cells to be sorted from the tumor prior to measurement of secretion, which could alter their functional behavior. Real time *in vivo* measurement of protein secretion would address both of these limitations, as well as provide a more dynamic picture of functional signaling for validation of scRNA-seq-derived cell-cell communication networks. As of yet, no real time *in vivo* methods have been demonstrated in the TME.

## “Pruning” Cell-Cell Communication Networks Computationally Narrows the Search for Experimentally Testable Targets

Another significant challenge when trying to reconstruct cell-cell communication networks from scRNA-seq data is to manage the inherent scale and complexity that these networks can attain. One approach is to use the aforementioned experimental approaches to collect spatial and protein-level information in order to restrict scRNA-seq-derived connections to those that can be experimentally confirmed. However, this can limit the potential of these computational inference methods to discover novel interactions. Because interaction networks are directed (i.e., unidirectional along each individual axis of communication), weighted (i.e., each axis has an associated score indicating whether the data supports the occurrence of that interaction), and densely connected, data mining and biological interpretation are complicated. However, they also present an opportunity to use concepts from graph theory and mathematical modeling to identify the most central, influential, or biologically relevant signaling axes for experimental validation.

Graph theory is a field of mathematics that views networks as a collection of nodes, or vertices, connected by edges. In the context of scRNA-seq-derived interaction networks, cell types or subtypes would be considered nodes, with ligand-receptor or ligand-target gene axes comprising the edges between them. Centrality is a graph theory concept concerned with identifying “important” nodes in a graph, where the definition of importance changes depending on the “angle” at which the graph is analyzed. Measures of centrality include degree, betweenness, closeness, pagerank, and eigenvalues. Degree is the simplest measure of centrality, counting the absolute number of edges pointing toward (i.e., in-degree) and away from (i.e., out-degree) a node. Betweenness quantifies a node’s influence on the flow of information in the network, which can identify “bottlenecks” or nodes that serve as “ambassadors” between modules of signaling. Similarly, closeness indicates how directly or indirectly each node in a graph can communicate with one another. Pagerank is a measure of “popularity,” as it answers the question of how often a particular node is communicated through during a random walk on the graph. Finally, eigenvalue centrality is similar to degree centrality in that it quantifies a node’s influence through its connections with others. However, this metric is better suited to a weighted network, as it takes strength of communication and influence of other nodes into account.

While centrality metrics are often reserved for analyzing scRNA-seq-derived gene regulatory networks (3, 147), they are becoming a useful tool to evaluate cell-cell communication. Raredon et al. (74) leveraged degree and eigenvector centralities, in addition to Kleinberg hub and authority scores, to quantitatively compare scRNA-seq-derived interaction networks from adult mammalian lungs across species. The Kleinberg hub and authority scores (148) were developed as extensions of eigenvector centrality for directed networks, where a “hub” is a node that communicates to other important nodes, and an “authority” is a node that is communicated to by other important nodes. With these measures of node centrality, Raredon et al. were able to confirm conserved patterns in signaling topologies in lung tissue across species. For example, alveolar type I (ATI) cells were unexpectedly characterized as a major signaling hub in mouse, rat, pig, and human lung, particularly for growth factor-related signaling families including vascular endothelial growth factor (VEGF) and semaphorins (SEMA). This work will lay the foundation for recapitulating homeostatic cell-cell communication in engineered lung tissue. A similar approach has yet to be applied to scRNA-seq from the TME, although this analysis is nearly directly transferable. Instead of revealing conserved mechanisms of signaling, comparing network centralities across homeostatic and cancerous conditions could identify changes in signaling topologies that give rise to aberrant communication underlying tumor growth, metastasis, and immune evasion. User-friendly tools for the interpretation of scRNA-seq-derived interaction networks from the perspective of graph theory are starting to become more popular in the literature, including the aforementioned CellChat (55).

Outside of graph theory, regression modeling presents an opportunity to interpret scRNA-seq-derived cell-cell communication networks with respect to relevant quantitative and qualitative biological behaviors, including tumor outcome, tumor growth rate, and immune cell infiltration. Elastic net regularization (149) is a regression modeling method that estimates the relationship between one or more features, or predictors, and a dependent outcome of interest, while accounting for overfitting and the instability imposed by high-dimensional data with few examples. Features are assigned coefficients representative of their relevance with respect to the outcome of interest, with coefficients for less predictive features shrinking to zero. Elastic net regularization has been previously applied to scRNA-seq to generate predictive gene signatures of immune cell identity (150), with the features in the regression problem being each gene in the transcriptome. Similarly, with features as ligand-receptor or ligand-target gene axis edges instead of genes, elastic net regularization could be used to select signatures of signaling predictive of aberrant or immunotherapy-altered tumor behaviors. This analysis would further filter network edges considered irrelevant in the context of the biological outcome of interest. Similar regression modeling approaches like Partial Least Squares Discriminant Analysis (PLS-DA) (151, 152) could further disentangle the directionality of the relationship between predictive signaling axes and emergent behaviors in the tumor. Overall, computational “pruning” strategies from graph theory and mathematical modeling are underappreciated and under-utilized resources for the distillation and interpretation of hypothesized interaction networks of the TME.

## Conclusion

scRNA-seq has proven to be a powerful tool for high throughput prediction of cell-cell communication. Transcriptome-wide profiling of genes encoding for ligands, receptors, intermediary signaling proteins, and signaling targets, in conjunction with novel algorithms like CellPhoneDB, CellChat, NicheNet, and CytoTalk, has characterized signaling mechanisms underlying homeostasis and disease across biological systems. In particular, scRNA-seq-derived interaction networks have facilitated identification of immunosuppressive and immunotherapy-altered cell-cell signaling in the TME, which contribute to the design and development of more effective immunotherapeutic interventions in the clinic. Validation of these networks will only serve to strengthen the hypotheses that they generate. Integration with SRT and other imaging technologies will help to define which interactions are physically possible in the microenvironment through co-localization of both communicating cell types and paired ligand, receptor, and signaling intermediaries. Functional measurement of protein secretion will confirm outgoing communication from “sender” cells and organize broader cell populations into relevant subsets based on patterns in secreted signaling. Further computational analysis of interaction networks by borrowing from the fields of mathematics and statistics will focus the search for experimentally targetable signaling mechanisms underlying aberrant behaviors. Together, integration of scRNA-seq-derived communication networks with powerful experimental and computational tools will expand our understanding of the role that cell-cell communication plays in emergent biological behaviors in the TME and beyond, improving outcomes for patients across malignancies.

## Author Contributions

KB and KM-J contributed equally to all aspects of this article. All authors read and approved the final manuscript.

## Funding

This work was supported by the U.S. National Institutes of Health (U01CA238728 to KM-J).

## Conflict of Interest

The authors declare that the research was conducted in the absence of any commercial or financial relationships that could be construed as a potential conflict of interest.

## Publisher’s Note

All claims expressed in this article are solely those of the authors and do not necessarily represent those of their affiliated organizations, or those of the publisher, the editors and the reviewers. Any product that may be evaluated in this article, or claim that may be made by its manufacturer, is not guaranteed or endorsed by the publisher.

## Footnotes

1
https://blog.wellcomeopenresearch.org/2020/07/17/to-share-and-share-alike-why-cellphonedb-a-friendly-open-source-platform-is-good-for-data-sharing-and-collaborations/

2
https://github.com/ventolab/CellphoneDB/blob/master/in/endometrium_atlas_example/endometrium_example_microenviroments.tsv

3
https://investors.isoplexis.com/news-releases/news-release-details/isoplexis-reports-preliminary-full-year-2021-revenue-releases

## References

[1] Wu AR Neff NF Kalisky T Dalerba P Treutlein B Rothenberg ME. Quantitative Assessment of Single-Cell RNA-Sequencing Methods. Nat Methods (2014) 11(1):41–6. doi: 10.1038/nmeth.2694 PMC4022966 24141493

[2] Buettner F Natarajan KN Casale FP Proserpio V Scialdone A Theis FJ. Computational Analysis of Cell-to-Cell Heterogeneity in Single-Cell RNA-Sequencing Data Reveals Hidden Subpopulations of Cells. Nat Biotechnol (2015) 33(2):155–60. doi: 10.1038/nbt.3102 25599176

[3] Iacono G Massoni-Badosa R Heyn H. Single-Cell Transcriptomics Unveils Gene Regulatory Network Plasticity. Genome Biol (2019) 20(1):110. doi: 10.1186/s13059-019-1713-4 31159854 PMC6547541

[4] Pal B Chen Y Vaillant F Jamieson P Gordon L Rios AC. Construction of Developmental Lineage Relationships in the Mouse Mammary Gland by Single-Cell RNA Profiling. Nat Commun (2017) 8(1):1627. doi: 10.1038/s41467-017-01560-x 29158510 PMC5696379

[5] Labani-Motlagh A Ashja-Mahdavi M Loskog A. The Tumor Microenvironment: A Milieu Hindering and Obstructing Antitumor Immune Responses. Front Immunol (2020) 11:940. doi: 10.3389/fimmu.2020.00940 32499786 PMC7243284

[6] Baghban R Roshangar L Jahanban-Esfahlan R Seidi K Ebrahimi-Kalan A Jaymand M. Tumor Microenvironment Complexity and Therapeutic Implications at a Glance. Cell Commun Signal (2020) 18(1):59. doi: 10.1186/s12964-020-0530-4 32264958 PMC7140346

[7] Anderson NM Simon MC. The Tumor Microenvironment. Curr Biol CB (2020) 30(16):R921–5. doi: 10.1016/j.cub.2020.06.081 PMC8194051 32810447

[8] Gajewski TF Schreiber H Fu Y-X. Innate and Adaptive Immune Cells in the Tumor Microenvironment. Nat Immunol (2013) 14(10):1014–22. doi: 10.1038/ni.2703 PMC4118725 24048123

[9] Kondo M. Lymphoid and Myeloid Lineage Commitment in Multipotent Hematopoietic Progenitors. Immunol Rev (2010) 238(1):37–46. doi: 10.1111/j.1600-065X.2010.00963.x 20969583 PMC2975965

[10] Vivier E Raulet DH Moretta A Caligiuri MA Zitvogel L Lanier LL. Innate or Adaptive Immunity? The Example of Natural Killer Cells. Science (2011) 331(6013):44–9. doi: 10.1126/science.1198687 PMC3089969 21212348

[11] Hinshaw DC Shevde LA. The Tumor Microenvironment Innately Modulates Cancer Progression. Cancer Res (2019) 79(18):4557–66. doi: 10.1158/0008-5472.CAN-18-3962 PMC6744958 31350295

[12] Chang RB Beatty GL. The Interplay Between Innate and Adaptive Immunity in Cancer Shapes the Productivity of Cancer Immunosurveillance. J Leukoc Biol (2020) 108(1):363–76. doi: 10.1002/JLB.3MIR0320-475R PMC7377219 32272502

[13] Pollard JW. Tumour-Educated Macrophages Promote Tumour Progression and Metastasis. Nat Rev Cancer. (2004) 4(1):71–8. doi: 10.1038/nrc1256 14708027

[14] Ruffell B Chang-Strachan D Chan V Rosenbusch A Ho CMT Pryer N. Macrophage IL-10 Blocks CD8+ T Cell-Dependent Responses to Chemotherapy by Suppressing IL-12 Expression in Intratumoral Dendritic Cells. Cancer Cell (2014) 26(5):623–37. doi: 10.1016/j.ccell.2014.09.006 PMC4254570 25446896

[15] He X Xu C. Immune Checkpoint Signaling and Cancer Immunotherapy. Cell Res (2020) 30(8):660–9. doi: 10.1038/s41422-020-0343-4 PMC7395714 32467592

[16] Joyce JA Fearon DT. T Cell Exclusion, Immune Privilege, and the Tumor Microenvironment. Science (2015) 348(6230):74–80. doi: 10.1126/science.aaa6204 25838376

[17] Chen DS Mellman I. Elements of Cancer Immunity and the Cancer–Immune Set Point. Nature (2017) 541(7637):321–30. doi: 10.1038/nature21349 28102259

[18] Yang Y. Cancer Immunotherapy: Harnessing the Immune System to Battle Cancer. J Clin Invest. (2015) 125(9):3335–7. doi: 10.1172/JCI83871 PMC4588312 26325031

[19] Esfahani K Roudaia L Buhlaiga N Del Rincon SV Papneja N Miller WH. A Review of Cancer Immunotherapy: From the Past, to the Present, to the Future. Curr Oncol (2020) 27(Suppl 2):S87–97. doi: 10.3747/co.27.5223 PMC7194005 32368178

[20] Pasquini G Rojo Arias JE Schäfer P Busskamp V. Automated Methods for Cell Type Annotation on scRNA-Seq Data. Comput Struct Biotechnol J (2021) 19:961–9. doi: 10.1016/j.csbj.2021.01.015 PMC7873570 33613863

[21] Cao X Xing L Majd E He H Gu J Zhang X. A Systematic Evaluation of Methods for Cell Phenotype Classification Using Single-Cell RNA Sequencing Data (2021). Available at: https://arxiv.org/abs/2110.00681v1. 10.3389/fgene.2022.836798 PMC8905542 35281805

[22] Abdelaal T Michielsen L Cats D Hoogduin D Mei H Reinders MJT. A Comparison of Automatic Cell Identification Methods for Single-Cell RNA Sequencing Data. Genome Biol (2019) 20(1):194. doi: 10.1186/s13059-019-1795-z 31500660 PMC6734286

[23] Huang Q Liu Y Du Y Garmire LX. Evaluation of Cell Type Annotation R Packages on Single-Cell RNA-Seq Data. Genomics Proteomics Bioinf (2021) 19(2):267–81. doi: 10.1016/j.gpb.2020.07.004 PMC8602772 33359678

[24] Zhou JX Taramelli R Pedrini E Knijnenburg T Huang S. Extracting Intercellular Signaling Network of Cancer Tissues Using Ligand-Receptor Expression Patterns From Whole-Tumor and Single-Cell Transcriptomes. Sci Rep (2017) 7(1):8815. doi: 10.1038/s41598-017-09307-w 28821810 PMC5562796

[25] Tirosh I Izar B Prakadan SM Wadsworth MH Treacy D Trombetta JJ. Dissecting the Multicellular Ecosystem of Metastatic Melanoma by Single-Cell RNA-Seq. Science (2016) 352(6282):189–96. doi: 10.1126/science.aad0501 PMC4944528 27124452

[26] Kumar MP Du J Lagoudas G Jiao Y Sawyer A Drummond DC. Analysis of Single-Cell RNA-Seq Identifies Cell-Cell Communication Associated With Tumor Characteristics. Cell Rep (2018) 25(6):1458–68. doi: 10.1016/j.celrep.2018.10.047 PMC7009724 30404002

[27] Ramilowski JA Goldberg T Harshbarger J Kloppmann E Lizio M Satagopam VP. A Draft Network of Ligand–Receptor-Mediated Multicellular Signalling in Human. Nat Commun (2015) 6(1):7866. doi: 10.1038/ncomms8866 26198319 PMC4525178

[28] Vento-Tormo R Efremova M Botting RA Turco MY Vento-Tormo M Meyer KB. Single-Cell Reconstruction of the Early Maternal–Fetal Interface in Humans. Nature (2018) 563(7731):347–53. doi: 10.1038/s41586-018-0698-6 PMC7612850 30429548

[29] Efremova M Vento-Tormo M Teichmann SA Vento-Tormo R. CellPhoneDB: Inferring Cell–Cell Communication From Combined Expression of Multi-Subunit Ligand–Receptor Complexes. Nat Protoc (2020) 15(4):1484–506. doi: 10.1038/s41596-020-0292-x 32103204

[30] The UniProt Consortium. UniProt: A Hub for Protein Information. Nucleic Acids Res (2015) 43(D1):D204–12. doi: 10.1093/nar/gku989 PMC4384041 25348405

[31] The UniProt Consortium. UniProt: The Universal Protein Knowledgebase in 2021. Nucleic Acids Res (2021) 49(D1):D480–9. doi: 10.1093/nar/gkaa1100 PMC7778908 33237286

[32] Hubbard T Barker D Birney E Cameron G Chen Y Clark L. The Ensembl Genome Database Project. Nucleic Acids Res (2002) 30(1):38–41. doi: 10.1093/nar/30.1.38 11752248 PMC99161

[33] Berman H Henrick K Nakamura H. Announcing the Worldwide Protein Data Bank. Nat Struct Mol Biol (2003) 10(12):980–0. doi: 10.1038/nsb1203-980 14634627

[34] wwPDB consortium. Protein Data Bank: The Single Global Archive for 3D Macromolecular Structure Data. Nucleic Acids Res (2019) 47(D1):D520–8. doi: 10.1093/nar/gky949 PMC6324056 30357364

[35] Orchard S Kerrien S Abbani S Aranda B Bhate J Bidwell S. Protein Interaction Data Curation: The International Molecular Exchange (IMEx) Consortium. Nat Methods (2012) 9(4):345–50. doi: 10.1038/nmeth.1931 PMC3703241 22453911

[36] Harmar AJ Hills RA Rosser EM Jones M Buneman OP Dunbar DR. IUPHAR-DB: The IUPHAR Database of G Protein-Coupled Receptors and Ion Channels. Nucleic Acids Res (2009) 37:D680–685. doi: 10.1093/nar/gkn728 PMC2686540 18948278

[37] Sharman JL Mpamhanga CP Spedding M Germain P Staels B Dacquet C. IUPHAR-DB: New Receptors and Tools for Easy Searching and Visualization of Pharmacological Data. Nucleic Acids Res (2011) 39:D534–8. doi: 10.1093/nar/gkq1062 PMC3013670 21087994

[38] Armstrong JF Faccenda E Harding SD Pawson AJ Southan C Sharman JL. The IUPHAR/BPS Guide to PHARMACOLOGY in 2020: Extending Immunopharmacology Content and Introducing the IUPHAR/MMV Guide to MALARIA PHARMACOLOGY. Nucleic Acids Res (2020) 48(D1):D1006–21. doi: 10.1093/nar/gkz951 PMC7145572 31691834

[39] Ricketts TD Prieto-Dominguez N Gowda PS Ubil E. Mechanisms of Macrophage Plasticity in the Tumor Environment: Manipulating Activation State to Improve Outcomes. Front Immunol (2021) 12:1565. doi: 10.3389/fimmu.2021.642285 PMC8139576 34025653

[40] Liu L Zhang R Deng J Dai X Zhu X Fu Q. Construction of TME and Identification of Crosstalk Between Malignant Cells and Macrophages by SPP1 in Hepatocellular Carcinoma. Cancer Immunol Immunother (2021) 71:121–36. doi: 10.1007/s00262-021-02967-8 PMC10992184 34028567

[41] Chen Z Zhao M Liang J Hu Z Huang Y Li M. Dissecting the Single-Cell Transcriptome Network Underlying Esophagus non-Malignant Tissues and Esophageal Squamous Cell Carcinoma. EBioMedicine (2021) 69:103459. doi: 10.1016/j.ebiom.2021.103459 34192657 PMC8253912

[42] Klement JD Paschall AV Redd PS Ibrahim ML Lu C Yang D. An Osteopontin/CD44 Immune Checkpoint Controls CD8+ T Cell Activation and Tumor Immune Evasion. J Clin Invest. (2018) 128(12):5549–60. doi: 10.1172/JCI123360 PMC6264631 30395540

[43] Mytar B Wołoszyn M Szatanek R Baj-Krzyworzeka M Siedlar M Ruggiero I. Tumor Cell-Induced Deactivation of Human Monocytes. J Leukoc Biol (2003) 74(6):1094–101. doi: 10.1189/jlb.0403140 12960282

[44] Zhang L Li Z Skrzypczynska KM Fang Q Zhang W O’Brien SA. Single-Cell Analyses Inform Mechanisms of Myeloid-Targeted Therapies in Colon Cancer. Cell (2020) 181(2):442–459.e29. doi: 10.1016/j.cell.2020.03.048 32302573

[45] Hargadon KM Johnson CE Williams CJ. Immune Checkpoint Blockade Therapy for Cancer: An Overview of FDA-Approved Immune Checkpoint Inhibitors. Int Immunopharmacol (2018) 62:29–39. doi: 10.1016/j.intimp.2018.06.001 29990692

[46] Wu X Gu Z Chen Y Chen B Chen W Weng L. Application of PD-1 Blockade in Cancer Immunotherapy. Comput Struct Biotechnol J (2019) 17:661–74. doi: 10.1016/j.csbj.2019.03.006 PMC6558092 31205619

[47] Bassez A Vos H Van Dyck L Floris G Arijs I Desmedt C. A Single-Cell Map of Intratumoral Changes During Anti-PD1 Treatment of Patients With Breast Cancer. Nat Med (2021) 27(5):820–32. doi: 10.1038/s41591-021-01323-8 33958794

[48] Bi K He MX Bakouny Z Kanodia A Napolitano S Wu J. Tumor and Immune Reprogramming During Immunotherapy in Advanced Renal Cell Carcinoma. Cancer Cell (2021) 39(5):649–61.e5. doi: 10.1016/j.ccell.2021.02.015 PMC8115394 33711272

[49] Jiang Y-Q Wang Z-X Zhong M Shen L-J Han X Zou X. Investigating Mechanisms of Response or Resistance to Immune Checkpoint Inhibitors by Analyzing Cell-Cell Communications in Tumors Before and After Programmed Cell Death-1 (PD-1) Targeted Therapy: An Integrative Analysis Using Single-Cell RNA and Bulk-RNA Sequencing Data. Oncoimmunology (2021) 10(1):1908010. doi: 10.1080/2162402X.2021.1908010 33868792 PMC8023241

[50] Das M Zhu C Kuchroo VK. Tim-3 and its Role in Regulating Anti-Tumor Immunity. Immunol Rev (2017) 276(1):97–111. doi: 10.1111/imr.12520 28258697 PMC5512889

[51] Hou R Denisenko E Ong HT Ramilowski JA Forrest ARR. Predicting Cell-to-Cell Communication Networks Using NATMI. Nat Commun (2020) 11(1):5011. doi: 10.1038/s41467-020-18873-z 33024107 PMC7538930

[52] Suzuki SR Kuno A Ozaki H. Cell-To-Cell Interaction Analysis of Prognostic Ligand-Receptor Pairs in Human Pancreatic Ductal Adenocarcinoma. Biochem Biophys Rep (2021) 28:101126. doi: 10.1016/j.bbrep.2021.101126 34522794 PMC8426203

[53] Cillo AR Kürten CH Tabib T Qi Z Onkar S Wang T. Immune Landscape of Viral- and Carcinogen-Driven Head and Neck Cancer. Immunity (2020) 52(1):183–199.e9. doi: 10.1016/j.immuni.2019.11.014 31924475 PMC7201194

[54] Noël F Massenet-Regad L Carmi-Levy I Cappuccio A Grandclaudon M Trichot C. Dissection of Intercellular Communication Using the Transcriptome-Based Framework ICELLNET. Nat Commun (2021) 12(1):1089. doi: 10.1038/s41467-021-21244-x 33597528 PMC7889941

[55] Jin S Guerrero-Juarez CF Zhang L Chang I Ramos R Kuan C-H. Inference and Analysis of Cell-Cell Communication Using CellChat. Nat Commun (2021) 12(1):1088. doi: 10.1038/s41467-021-21246-9 33597522 PMC7889871

[56] Szklarczyk D Gable AL Lyon D Junge A Wyder S Huerta-Cepas J. STRING V11: Protein-Protein Association Networks With Increased Coverage, Supporting Functional Discovery in Genome-Wide Experimental Datasets. Nucleic Acids Res (2019) 47(D1):D607–13. doi: 10.1093/nar/gky1131 PMC6323986 30476243

[57] Cowen L Ideker T Raphael BJ Sharan R. Network Propagation: A Universal Amplifier of Genetic Associations. Nat Rev Genet (2017) 18(9):551–62. doi: 10.1038/nrg.2017.38 28607512

[58] Lee AH Sun L Mochizuki AY Reynoso JG Orpilla J Chow F. Neoadjuvant PD-1 Blockade Induces T Cell and Cdc1 Activation But Fails to Overcome the Immunosuppressive Tumor Associated Macrophages in Recurrent Glioblastoma. Nat Commun (2021) 12(1):6938. doi: 10.1038/s41467-021-26940-2 34836966 PMC8626557

[59] Yin H Guo R Zhang H Liu S Gong Y Yuan Y. A Dynamic Transcriptome Map of Different Tissue Microenvironment Cells Identified During Gastric Cancer Development Using Single-Cell RNA Sequencing. Front Immunol (2021) 12:728169. doi: 10.3389/fimmu.2021.728169 34745098 PMC8566821

[60] Park Y Jeong J Seong S Kim W. In Silico Evaluation of Natural Compounds for an Acidic Extracellular Environment in Human Breast Cancer. Cells (2021) 10(10):2673. doi: 10.3390/cells10102673 34685653 PMC8534855

[61] Wang Y Wang R Zhang S Song S Jiang C Han G. iTALK: An R Package to Characterize and Illustrate Intercellular Communicatio. (2019). doi: 10.1101/507871.

[62] Cabello-Aguilar S Alame M Kon-Sun-Tack F Fau C Lacroix M Colinge J. SingleCellSignalR: Inference of Intercellular Networks From Single-Cell Transcriptomics. Nucleic Acids Res (2020) 48(10):e55. doi: 10.1093/nar/gkaa183 32196115 PMC7261168

[63] Garcia-Alonso L Handfield L-F Roberts K Nikolakopoulou K Fernando RC Gardner L. Mapping the Temporal and Spatial Dynamics of the Human Endometrium In Vivo and *In Vitro* . Nat Genet (2021) 53(12):1698–711. doi: 10.1038/s41588-021-00972-2 PMC8648563 34857954

[64] Browaeys R Saelens W Saeys Y. NicheNet: Modeling Intercellular Communication by Linking Ligands to Target Genes. Nat Methods (2020) 17(2):159–62. doi: 10.1038/s41592-019-0667-5 31819264

[65] Hu Y Peng T Gao L Tan K. CytoTalk: De Novo Construction of Signal Transduction Networks Using Single-Cell Transcriptomic Data. Sci Adv (2021) 7(16):eabf1356. doi: 10.1126/sciadv.abf1356 33853780 PMC8046375

[66] Cheng S Li Z Gao R Xing B Gao Y Yang Y. A Pan-Cancer Single-Cell Transcriptional Atlas of Tumor Infiltrating Myeloid Cells. Cell (2021) 184(3):792–809.e23. doi: 10.1016/j.cell.2021.01.010 33545035

[67] Maier B Leader AM Chen ST Tung N Chang C LeBerichel J. A Conserved Dendritic-Cell Regulatory Program Limits Antitumour Immunity. Nature (2020) 580(7802):257–62. doi: 10.1038/s41586-020-2134-y PMC7787191 32269339

[68] Ribatti D. Mast Cells as Therapeutic Target in Cancer. Eur J Pharmacol (2016) 778:152–7. doi: 10.1016/j.ejphar.2015.02.056 25917325

[69] Türei D Korcsmáros T Saez-Rodriguez J. OmniPath: Guidelines and Gateway for Literature-Curated Signaling Pathway Resources. Nat Methods (2016) 13(12):966–7. doi: 10.1038/nmeth.4077 27898060

[70] Bianchi JJ Zhao X Mays JC Davoli T. Not All Cancers are Created Equal: Tissue Specificity in Cancer Genes and Pathways. Curr Opin Cell Biol (2020) 63:135–43. doi: 10.1016/j.ceb.2020.01.005 PMC7247947 32092639

[71] Graeber TG Eisenberg D. Bioinformatic Identification of Potential Autocrine Signaling Loops in Cancers From Gene Expression Profiles. Nat Genet (2001) 29(3):295–300. doi: 10.1038/ng755 11685206

[72] Ben-Shlomo I Yu Hsu S Rauch R Kowalski HW Hsueh AJW. Signaling Receptome: A Genomic and Evolutionary Perspective of Plasma Membrane Receptors Involved in Signal Transduction. Sci STKE Signal Transduct Knowl Environ (2003) 2003(187):RE9. doi: 10.1126/stke.2003.187.re9 12815191

[73] Puram SV Tirosh I Parikh AS Patel AP Yizhak K Gillespie S. Single-Cell Transcriptomic Analysis of Primary and Metastatic Tumor Ecosystems in Head and Neck Cancer. Cell (2017) 171(7):1611–1624.e24. doi: 10.1016/j.cell.2017.10.044 29198524 PMC5878932

[74] Raredon MSB Adams TS Suhail Y Schupp JC Poli S Neumark N. Single-Cell Connectomic Analysis of Adult Mammalian Lungs. Sci Adv (2019) 5(12):eaaw3851. doi: 10.1126/sciadv.aaw3851 31840053 PMC6892628

[75] Baccin C Al-Sabah J Velten L Helbling PM Grünschläger F Hernández-Malmierca P. Combined Single-Cell and Spatial Transcriptomics Reveal the Molecular, Cellular and Spatial Bone Marrow Niche Organization. Nat Cell Biol (2020) 22(1):38–48. doi: 10.1038/s41556-019-0439-6 31871321 PMC7610809

[76] Oughtred R Rust J Chang C Breitkreutz B Stark C Willems A. The BioGRID Database: A Comprehensive Biomedical Resource of Curated Protein, Genetic, and Chemical Interactions. Protein Sci Publ Protein Soc (2021) 30(1):187–200. doi: 10.1002/pro.3978 PMC7737760 33070389

[77] Jassal B Matthews L Viteri G Gong C Lorente P Fabregat A. The Reactome Pathway Knowledgebase. Nucleic Acids Res (2020) 48(D1):D498–503. doi: 10.1093/nar/gkz1031 PMC7145712 31691815

[78] Kanehisa M Furumichi M Tanabe M Sato Y Morishima K. KEGG: New Perspectives on Genomes, Pathways, Diseases and Drugs. Nucleic Acids Res (2017) 45(D1):D353–61. doi: 10.1093/nar/gkw1092 PMC5210567 27899662

[79] Zhao Q-Y Liu L-P Lu L Gui R Luo Y-W. A Novel Intercellular Communication-Associated Gene Signature for Prognostic Prediction and Clinical Value in Patients With Lung Adenocarcinoma. Front Genet (2021) 12:702424. doi: 10.3389/fgene.2021.702424 34497634 PMC8419521

[80] Griffith JW Sokol CL Luster AD. Chemokines and Chemokine Receptors: Positioning Cells for Host Defense and Immunity. Annu Rev Immunol (2014) 32:659–702. doi: 10.1146/annurev-immunol-032713-120145 24655300

[81] Auslander N Zhang G Lee JS Frederick DT Miao B Moll T. Robust Prediction of Response to Immune Checkpoint Blockade Therapy in Metastatic Melanoma. Nat Med (2018) 24(10):1545–9. doi: 10.1038/s41591-018-0157-9 PMC6693632 30127394

[82] Cameron MJ Kelvin DJ. Cytokines and Chemokines–Their Receptors and Their Genes: An Overview. Adv Exp Med Biol (2003) 520:8–32. doi: 10.1007/978-1-4615-0171-8_2 12613570

[83] Bao X Zhang H Wu W Cheng S Dai X Zhu X. Analysis of the Molecular Nature Associated With Microsatellite Status in Colon Cancer Identifies Clinical Implications for Immunotherapy. J Immunother Cancer (2020) 8(2):e001437. doi: 10.1136/jitc-2020-001437 33028695 PMC7542666

[84] Telli ML Nagata H Wapnir I Acharya CR Zablotsky K Fox BA. Intratumoral Plasmid IL12 Expands CD8+ T Cells and Induces a CXCR3 Gene Signature in Triple-Negative Breast Tumors That Sensitizes Patients to Anti–PD-1 Therapy. Clin Cancer Res (2021) 27(9):2481–93. doi: 10.1158/1078-0432.CCR-20-3944 PMC8102331 33593880

[85] Smalley I Chen Z Phadke M Li J Yu X Wyatt C. Single-Cell Characterization of the Immune Microenvironment of Melanoma Brain and Leptomeningeal Metastases. Clin Cancer Res (2021) 27(14):4109–25. doi: 10.1158/1078-0432.CCR-21-1694 PMC8282775 34035069

[86] Prasad TSK Kandasamy K Pandey A. Human Protein Reference Database and Human Proteinpedia as Discovery Tools for Systems Biology. Methods Mol Biol Clifton NJ (2009) 577:67–79. doi: 10.1007/978-1-60761-232-2_6 19718509

[87] Dimmer EC Huntley RP Alam-Faruque Y Sawford T O’Donovan C Martin MJ. The UniProt-GO Annotation Database in 2011. Nucleic Acids Res (2012) 40:D565–570. doi: 10.1093/nar/gkr1048 PMC3245010 22123736

[88] Subramanian A Tamayo P Mootha VK Mukherjee S Ebert BL Gillette MA. Gene Set Enrichment Analysis: A Knowledge-Based Approach for Interpreting Genome-Wide Expression Profiles. Proc Natl Acad Sci (2005) 102(43):15545–50. doi: 10.1073/pnas.0506580102 PMC1239896 16199517

[89] Armingol E Officer A Harismendy O Lewis NE. Deciphering Cell–Cell Interactions and Communication From Gene Expression. Nat Rev Genet (2021) 22(2):71–88. doi: 10.1038/s41576-020-00292-x 33168968 PMC7649713

[90] Ma F Zhang S Song L Wang B Wei L Zhang F. Applications and Analytical Tools of Cell Communication Based on Ligand-Receptor Interactions at Single Cell Level. Cell Biosci (2021) 11(1):121. doi: 10.1186/s13578-021-00635-z 34217372 PMC8254218

[91] Nguyen QH Pervolarakis N Nee K Kessenbrock K. Experimental Considerations for Single-Cell RNA Sequencing Approaches. Front Cell Dev Biol (2018) 6:108. doi: 10.3389/fcell.2018.00108 30234113 PMC6131190

[92] Yuan G-C Cai L Elowitz M Enver T Fan G Guo G. Challenges and Emerging Directions in Single-Cell Analysis. Genome Biol (2017) 18(1):84. doi: 10.1186/s13059-017-1218-y 28482897 PMC5421338

[93] Melero I Rouzaut A Motz GT Coukos G. T-Cell and NK-Cell Infiltration Into Solid Tumors: A Key Limiting Factor for Efficacious Cancer Immunotherapy. Cancer Discovery (2014) 4(5):522–6. doi: 10.1158/2159-8290.CD-13-0985 PMC4142435 24795012

[94] Gubin MM Esaulova E Ward JP Malkova ON Runci D Wong P. High-Dimensional Analysis Delineates Myeloid and Lymphoid Compartment Remodeling During Successful Immune-Checkpoint Cancer Therapy. Cell (2018) 175(4):1014–1030.e19. doi: 10.1016/j.cell.2018.09.030 30343900 PMC6501221

[95] Zilionis R Engblom C Pfirschke C Savova V Zemmour D Saatcioglu HD. Single-Cell Transcriptomics of Human and Mouse Lung Cancers Reveals Conserved Myeloid Populations Across Individuals and Species. Immunity (2019) 50(5):1317–1334.e10. doi: 10.1016/j.immuni.2019.03.009 30979687 PMC6620049

[96] Alshetaiwi H Pervolarakis N McIntyre LL Ma D Nguyen Q Rath JA. Defining the Emergence of Myeloid-Derived Suppressor Cells in Breast Cancer Using Single-Cell Transcriptomics. Sci Immunol (2020) 115(44):eaay6017. doi: 10.1126/sciimmunol.aay6017 PMC7219211 32086381

[97] Mucci A Antonarelli G Caserta C Vittoria FM Desantis G Pagani R. Myeloid Cell-Based Delivery of IFN-γ Reprograms the Leukemia Microenvironment and Induces Anti-Tumoral Immune Responses. EMBO Mol Med (2021) 13(10):e13598. doi: 10.15252/emmm.202013598 34459560 PMC8495462

[98] Perry CJ Muñoz-Rojas AR Meeth KM Kellman LN Amezquita RA Thakral D. Myeloid-Targeted Immunotherapies Act in Synergy to Induce Inflammation and Antitumor Immunity. J Exp Med (2018) 215(3):877–93. doi: 10.1084/jem.20171435 PMC5839759 29436395

[99] Pai SI Cesano A Marincola FM. The Paradox of Cancer Immune Exclusion: Immune Oncology Next Frontier. Cancer Treat Res (2020) 180:173–95. doi: 10.1007/978-3-030-38862-1_6 PMC7423459 32215870

[100] Berglund E Maaskola J Schultz N Friedrich S Marklund M Bergenstråhle J. Spatial Maps of Prostate Cancer Transcriptomes Reveal an Unexplored Landscape of Heterogeneity. Nat Commun (2018) 9(1):2419. doi: 10.1038/s41467-018-04724-5 29925878 PMC6010471

[101] Thrane K Eriksson H Maaskola J Hansson J Lundeberg J. Spatially Resolved Transcriptomics Enables Dissection of Genetic Heterogeneity in Stage III Cutaneous Malignant Melanoma. Cancer Res (2018) 78(20):5970–9. doi: 10.1158/0008-5472.CAN-18-0747 30154148

[102] Marx V. Method of the Year: Spatially Resolved Transcriptomics. Nat Methods (2021) 18(1):9–14. doi: 10.1038/s41592-020-01033-y 33408395

[103] Maniatis S Petrescu J Phatnani H. Spatially Resolved Transcriptomics and its Applications in Cancer. Curr Opin Genet Dev (2021) 1;66:70–7. doi: 10.1016/j.gde.2020.12.002 PMC7969406 33434721

[104] Rozenblatt-Rosen O Stubbington MJT Regev A Teichmann SA. The Human Cell Atlas: From Vision to Reality. Nature (2017) 550(7677):451–3. doi: 10.1038/550451a 29072289

[105] Asp M Bergenstråhle J Lundeberg J. Spatially Resolved Transcriptomes—Next Generation Tools for Tissue Exploration. BioEssays (2020) 42(10):1900221. doi: 10.1002/bies.201900221 32363691

[106] Wang N Li X Wang R Ding Z. Spatial Transcriptomics and Proteomics Technologies for Deconvoluting the Tumor Microenvironment. Biotechnol J (2021) 16(9):2100041. doi: 10.1002/biot.202100041 34125481

[107] Bassiouni R Gibbs LD Craig DW Carpten JD McEachron TA. Applicability of Spatial Transcriptional Profiling to Cancer Research. Mol Cell (2021) 81(8):1631–9. doi: 10.1016/j.molcel.2021.03.016 PMC8052283 33826920

[108] Moncada R Barkley D Wagner F Chiodin M Devlin JC Baron M. Integrating Microarray-Based Spatial Transcriptomics and Single-Cell RNA-Seq Reveals Tissue Architecture in Pancreatic Ductal Adenocarcinomas. Nat Biotechnol (2020) 38(3):333–42. doi: 10.1038/s41587-019-0392-8 31932730

[109] Ståhl PL Salmén F Vickovic S Lundmark A Navarro JF Magnusson J. Visualization and Analysis of Gene Expression in Tissue Sections by Spatial Transcriptomics. Science (2016) 353(6294):78–82. doi: 10.1126/science.aaf2403 27365449

[110] Chen KH Boettiger AN Moffitt JR Wang S Zhuang X. Spatially Resolved, Highly Multiplexed RNA Profiling in Single Cells. Science (2015) 348:6233. doi: 10.1126/science.aaa6090 PMC4662681 25858977

[111] Xia C Fan J Emanuel G Hao J Zhuang X. Spatial Transcriptome Profiling by MERFISH Reveals Subcellular RNA Compartmentalization and Cell Cycle-Dependent Gene Expression. Proc Natl Acad Sci (2019) 116(39):19490–9. doi: 10.1073/pnas.1912459116 PMC6765259 31501331

[112] Merritt CR Ong GT Church SE Barker K Danaher P Geiss G. Multiplex Digital Spatial Profiling of Proteins and RNA in Fixed Tissue. Nat Biotechnol (2020) 38(5):586–99. doi: 10.1038/s41587-020-0472-9 32393914

[113] Hara T Chanoch-Myers R Mathewson ND Myskiw C Atta L Bussema L. Interactions Between Cancer Cells and Immune Cells Drive Transitions to Mesenchymal-Like States in Glioblastoma. Cancer Cell (2021) 39(6):779–792.e11. doi: 10.1016/j.ccell.2021.05.002 34087162 PMC8366750

[114] Kim Y Varn FS Park S-H Yoon BW Park HR Lee C. Perspective of Mesenchymal Transformation in Glioblastoma. Acta Neuropathol Commun (2021) 9(1):50. doi: 10.1186/s40478-021-01151-4 33762019 PMC7992784

[115] Price C Chen J Pelka K Chao S He J Boland G. 920 A Single-Cell Spatially Resolved MERFISH Map of the Colorectal Tumor Immune Microenvironment. J Immunother Cancer (2021) 9(Suppl 2). doi: 10.1136/jitc-2021-SITC2021.920

[116] Toki MI Merritt CR Wong PF Smithy JW Kluger HM Syrigos KN. High-Plex Predictive Marker Discovery for Melanoma Immunotherapy-Treated Patients Using Digital Spatial Profiling. Clin Cancer Res Off J Am Assoc Cancer Res (2019) 25(18):5503–12. doi: 10.1158/1078-0432.CCR-19-0104 PMC6744974 31189645

[117] Zugazagoitia J Gupta S Liu Y Fuhrman K Gettinger S Herbst RS. Biomarkers Associated With Beneficial PD-1 Checkpoint Blockade in Non–Small Cell Lung Cancer (NSCLC) Identified Using High-Plex Digital Spatial Profiling. Clin Cancer Res (2020) 26(16):4360–8. doi: 10.1158/1078-0432.CCR-20-0175 PMC7442721 32253229

[118] Kulasinghe A Taheri T O’Byrne K Hughes BGM Kenny L Punyadeera C. Highly Multiplexed Digital Spatial Profiling of the Tumor Microenvironment of Head and Neck Squamous Cell Carcinoma Patients. Front Oncol (2021) 10:607349. doi: 10.3389/fonc.2020.607349 33542903 PMC7851078

[119] Liu Y Yang M Deng Y Su G Enninful A Guo CC. High-Spatial-Resolution Multi-Omics Sequencing *via* Deterministic Barcoding in Tissue. Cell (2020) 183(6):1665–1681.e18. doi: 10.1016/j.cell.2020.10.026 33188776 PMC7736559

[120] Eng C-HL Lawson M Zhu Q Dries R Koulena N Takei Y. Transcriptome-Scale Super-Resolved Imaging in Tissues by RNA seqFISH+. Nature (2019) 568(7751):235–9. doi: 10.1038/s41586-019-1049-y PMC6544023 30911168

[121] Rodriques SG Stickels RR Goeva A Martin CA Murray E Vanderburg CR. Slide-Seq: A Scalable Technology for Measuring Genome-Wide Expression at High Spatial Resolution. Science (2019) 363(6434):1463–7. doi: 10.1126/science.aaw1219 PMC6927209 30923225

[122] Miller MA Weissleder R. Imaging the Pharmacology of Nanomaterials by Intravital Microscopy: Towards Understanding Their Biological Behavior. Adv Drug Delivery Rev (2017) 113:61–86. doi: 10.1016/j.addr.2016.05.023 PMC5136524 27266447

[123] Luthria G Li R Wang S Prytyskach M Kohler RH Lauffenburger DA. *In Vivo* Microscopy Reveals Macrophage Polarization Locally Promotes Coherent Microtubule Dynamics in Migrating Cancer Cells. Nat Commun (2020) 11(1):3521. doi: 10.1038/s41467-020-17147-y 32665556 PMC7360550

[124] Garris CS Arlauckas SP Kohler RH Trefny MP Garren S Piot C. Successful Anti-PD-1 Cancer Immunotherapy Requires T Cell-Dendritic Cell Crosstalk Involving the Cytokines IFN-γ and IL-12. Immunity (2018) 49(6):1148–61.e7. doi: 10.1016/j.immuni.2018.09.024 30552023 PMC6301092

[125] Di Pilato M Kfuri-Rubens R Pruessmann JN Ozga AJ Messemaker M Cadilha BL. CXCR6 Positions Cytotoxic T Cells to Receive Critical Survival Signals in the Tumor Microenvironment. Cell (2021) 184(17):4512–30.e22. doi: 10.1016/j.cell.2021.07.015 34343496 PMC8719451

[126] Khoo WH Ledergor G Weiner A Roden DL Terry RL McDonald MM. A Niche-Dependent Myeloid Transcriptome Signature Defines Dormant Myeloma Cells. Blood (2019) 134(1):30–43. doi: 10.1182/blood.2018880930 31023703

[127] Lam FC Morton SW Wyckoff J Vu Han T-L Hwang MK Maffa A. Enhanced Efficacy of Combined Temozolomide and Bromodomain Inhibitor Therapy for Gliomas Using Targeted Nanoparticles. Nat Commun (2018) 9(1):1991. doi: 10.1038/s41467-018-04315-4 29777137 PMC5959860

[128] Yu X Chen L Liu J Dai B Xu G Shen G. Immune Modulation of Liver Sinusoidal Endothelial Cells by Melittin Nanoparticles Suppresses Liver Metastasis. Nat Commun (2019) 10(1):574. doi: 10.1038/s41467-019-08538-x 30718511 PMC6361944

[129] Cho C-F Yu L Nsiama TK Kadam AN Raturi A Shukla S. Viral Nanoparticles Decorated With Novel EGFL7 Ligands Enable Intravital Imaging of Tumor Neovasculature. Nanoscale (2017) 9(33):12096–109. doi: 10.1039/C7NR02558K PMC5770569 28799610

[130] Fisher DT Muhitch JB Kim M Doyen KC Bogner PN Evans SS. Intraoperative Intravital Microscopy Permits the Study of Human Tumour Vessels. Nat Commun (2016) 17 7:10684. doi: 10.1038/ncomms10684 PMC4757793 26883450

[131] Lin J-R Izar B Wang S Yapp C Mei S Shah PM. Highly Multiplexed Immunofluorescence Imaging of Human Tissues and Tumors Using T-CyCIF and Conventional Optical Microscopes Editors. eLife (2018) 7:e31657. doi: 10.7554/eLife.31657 PMC6075866 29993362

[132] Jerby-Arnon L Shah P Cuoco MS Rodman C Su M-J Melms JC. A Cancer Cell Program Promotes T Cell Exclusion and Resistance to Checkpoint Blockade. Cell (2018) 175(4):984–997.e24. doi: 10.1016/j.cell.2018.09.006 30388455 PMC6410377

[133] Mehta AK Cheney EM Hartl CA Pantelidou C Oliwa M Castrillon JA. Targeting Immunosuppressive Macrophages Overcomes PARP Inhibitor Resistance in BRCA1-Associated Triple-Negative Breast Cancer. Nat Cancer (2021) 2(1):66–82. doi: 10.1038/s43018-020-00148-7 33738458 PMC7963404

[134] Yu X Schneiderhan-Marra N Joos TO. Protein Microarrays for Personalized Medicine. Clin Chem (2010) 56(3):376–87. doi: 10.1373/clinchem.2009.137158 PMC7108201 20075183

[135] Ma C Fan R Ahmad H Shi Q Comin-Anduix B Chodon T. A Clinical Microchip for Evaluation of Single Immune Cells Reveals High Functional Heterogeneity in Phenotypically Similar T Cells. Nat Med (2011) 17(6):738–43. doi: 10.1038/nm.2375 PMC3681612 21602800

[136] Shi Q Qin L Wei W Geng F Fan R Shin YS. Single-Cell Proteomic Chip for Profiling Intracellular Signaling Pathways in Single Tumor Cells. Proc Natl Acad Sci (2012) 109(2):419–24. doi: 10.1073/pnas.1110865109 PMC3258586 22203961

[137] Zhao JL Ma C O’Connell RM Mehta A DiLoreto R Heath JR. Conversion of Danger Signals Into Cytokine Signals by Hematopoietic Stem and Progenitor Cells for Regulation of Stress-Induced Hematopoiesis. Cell Stem Cell (2014) 14(4):445–59. doi: 10.1016/j.stem.2014.01.007 PMC4119790 24561084

[138] Lu Y Chen JJ Mu L Xue Q Wu Y Wu P-H. High-Throughput Secretomic Analysis of Single Cells to Assess Functional Cellular Heterogeneity. Anal Chem (2013) 85(4):2548–56. doi: 10.1021/ac400082e PMC3589817 23339603

[139] Lu Y Xue Q Eisele MR Sulistijo ES Brower K Han L. Highly Multiplexed Profiling of Single-Cell Effector Functions Reveals Deep Functional Heterogeneity in Response to Pathogenic Ligands. Proc Natl Acad Sci (2015) 112(7):E607–15. doi: 10.1073/pnas.1416756112 PMC4343126 25646488

[140] Xue Q Lu Y Eisele MR Sulistijo ES Khan N Fan R. Analysis of Single-Cell Cytokine Secretion Reveals a Role for Paracrine Signaling in Coordinating Macrophage Responses to TLR4 Stimulation. Sci Signal (2015) 8(381):ra59. doi: 10.1126/scisignal.aaa2155 26082435 PMC5735825

[141] Alexander AF Kelsey I Forbes H Miller-Jensen K. Single-Cell Secretion Analysis Reveals a Dual Role for IL-10 in Restraining and Resolving the TLR4-Induced Inflammatory Response. Cell Rep (2021) 36(12):109728. doi: 10.1016/j.celrep.2021.109728 34551303 PMC8995750

[142] Henry CJ Ornelles DA Mitchell LM Brzoza-Lewis KL Hiltbold EM. IL-12 Produced by Dendritic Cells Augments CD8+ T Cell Activation Through the Production of the Chemokines CCL1 and CCL17. J Immunol Baltim Md (2008) 181(12):8576–84. doi: 10.4049/jimmunol.181.12.8576 PMC2716729 19050277

[143] Creelan BC Wang C Teer JK Toloza EM Yao J Kim S. Tumor-Infiltrating Lymphocyte Treatment for Anti-PD-1-Resistant Metastatic Lung Cancer: A Phase 1 Trial. Nat Med (2021) 27(8):1410–8. doi: 10.1038/s41591-021-01462-y PMC8509078 34385708

[144] Abbas HA Alaniz Z Mackay S Cyr M Zhou J Issa GC. Single-Cell Polyfunctional Proteomics of CD4 Cells From Patients With AML Predicts Responses to Anti–PD-1–Based Therapy. Blood Adv (2021) 5(22):4569–74. doi: 10.1182/bloodadvances.2021004583 PMC8759127 34555853

[145] Fousek K Watanabe J Joseph SK George A An X Byrd TT. CAR T-Cells That Target Acute B-Lineage Leukemia Irrespective of CD19 Expression. Leukemia (2021) 35(1):75–89. doi: 10.1038/s41375-020-0792-2 32205861 PMC7519582

[146] Spiegel JY Patel S Muffly L Hossain NM Oak J Baird JH. CAR T Cells With Dual Targeting of CD19 and CD22 in Adult Patients With Recurrent or Refractory B Cell Malignancies: A Phase 1 Trial. Nat Med (2021) 27(8):1419–31. doi: 10.1038/s41591-021-01436-0 PMC8363505 34312556

[147] Zhou S Huang Y Liu H Zhou X Yuan M Hou F. Single-Cell RNA-Seq Dissects the Intratumoral Heterogeneity of Triple-Negative Breast Cancer Based on Gene Regulatory Networks. Mol Ther - Nucleic Acids (2021) 5 23:682–90. doi: 10.1016/j.omtn.2020.12.018 PMC7851423 33575114

[148] Kleinberg JM. Authoritative Sources in a Hyperlinked Environment. J ACM. (1999) 46(5):604–32. doi: 10.1145/324133.324140

[149] Zou H Hastie T. Regularization and Variable Selection *via* the Elastic Net. J R Stat Soc Ser B Stat Methodol (2005) 67(2):301–20. doi: 10.1111/j.1467-9868.2005.00503.x

[150] Torang A Gupta P Klinke DJ. An Elastic-Net Logistic Regression Approach to Generate Classifiers and Gene Signatures for Types of Immune Cells and T Helper Cell Subsets. BMC Bioinf (2019) 20(1):433. doi: 10.1186/s12859-019-2994-z PMC6704630 31438843

[151] Barker M Rayens W. Partial Least Squares for Discrimination. J Chemom (2003) 17(3):166–73. doi: 10.1002/cem.785

[152] Ruiz-Perez D Guan H Madhivanan P Mathee K Narasimhan G. So You Think You can PLS-Da? BMC Bioinf (2020) 21(1):2. doi: 10.1186/s12859-019-3310-7 PMC7724830 33297937

